# Physical Zoo in Pb-Cu-P-S-O Apatite

**DOI:** 10.3390/ma18204728

**Published:** 2025-10-15

**Authors:** Hongyang Wang, Hao Wu, Yijing Zhao, Kun Tao, Zhixing Wu, Zhihui Geng, Tianbao Wang, Shufeng Ye, Ning Chen

**Affiliations:** 1 State Key Laboratory of Mesoscience and Engineering, Institute of Process Engineering, Chinese Academy of Sciences, Beijing 100190, China; sfye@ipe.ac.cn; 2 Shanghai ZhenKeAiDai Materials Co., Ltd., Shanghai 201600, China; woohaw1993@gmail.com (H.W.); georgiageorgiawang@163.com (Y.Z.); 231327192@fzu.edu.cn (Z.W.); geng.zhihui486@gmail.com (Z.G.);; 3 School of Materials Science and Engineering, Huazhong University of Science and Technology, Wuhan 430074, China; 4 College of Business Global Campus, University College Dublin, D04 V1W8 Dublin, Ireland; 5 School of Physical Science and Technology, Lanzhou University, Lanzhou 730000, China; taokun@lzu.edu.cn; 6 MOE Key Laboratory for Analytical Science of Food Safety and Biology, College of Chemistry, Fuzhou University, Fuzhou 350108, China; 7 College of Computer Science and Technology, Zhejiang University, Hangzhou 310007, China; 8 School of Materials Science and Engineering, University of Science and Technology Beijing, Beijing 100083, China; nchen@sina.com

**Keywords:** copper-doped lead apatite, possible near-room-temperature superconductivity, variable physical properties

## Abstract

**Highlights:**

**What are the main findings?**

- The formation of one-dimensional chain-like structures, through the co-doping of copper and an excess of non-metallic elements within the lead apatite framework, may be critical for achieving superconductivity.

**What is the implication of the main finding?**

- Doping of non-metallic elements in copper-doped lead apatite gives rise to a rich variety of physical phenomena within the system.
- The apatite family of materials holds significant potential as a key platform for advancing research on strongly correlated physics.

**Abstract:**

Further constraints on material dimensionality are expected to allow for the emergence of more physical phases. However, the thermal stability of materials tends to decrease in lower dimensions. The quasi-one-dimensional structure within apatite offers an ideal framework for doping. Using copper-doped lead apatite as the foundational structure, further doping with non-metallic elements can induce transitions between insulating, semiconducting, metallic, and even superconducting states, as well as giving rise to diverse magnetic properties. This effectively creates a veritable ’zoo of physics’.

## 1. Introduction

The strategic manipulation of non-metallic elements within materials to induce local electron or hole defects is increasingly recognized as a pivotal factor in achieving strange phenomena [1,2,3]. In August 2023, Lee, Kim et al. [4]. proposed apatite as a candidate system for high-temperature superconductivity, with a potential critical temperature ($T_{c}$) exceeding 300 K. This assertion ignited significant controversy [5]. In the ongoing quest for novel superconducting materials, apatite structures offer a compelling platform due to their readily solid framework and quasi-one-dimensional channels [6,7], metaphorically described as “wastebaskets” capable of accommodating various dopants. The excessive doping of apatite with non-metallic elements results in the development of polymeric chain structures and potential ionic bonds. Furthermore, the introduction of copper imparts magnetic atoms to the system [8]. Consequently, by systematically varying the doping ratio of sulfur (or oxygen) ions within the apatite channels, copper-doped lead apatite can be transformed into a series of complex compounds [9]. Firstly, the cationic sites within apatite are highly susceptible to substitution; copper doping can be conceptualized as an extension of conventional cuprate superconductors [10]. Secondly, the quasi-one-dimensional channels inherent in the apatite structure offer abundant sites for doping or defect engineering, facilitating straightforward electron or hole control [11,12]. Consequently, the structural characteristics of apatite position it as a promising platform for investigating novel physical phenomena [13].

However, over the subsequent months, numerous experimental efforts failed to replicate the claimed superconductivity. Ultimately, the phenomenon was attributed to a phase transition of Cu_2_S [14], effectively concluding the debate. Notwithstanding the complex physical mechanisms involved, from a materials science perspective, apatite remains a viable avenue for superconductivity research [15,16].

In the synthesis method of LK-99, the co-roasting of ${\mathrm{Cu}}_{3}$P and ${\mathrm{Pb}}_{2}$${\mathrm{SO}}_{5}$ presents a distinct scenario compared to conventional apatite synthesis, often resembling a stochastic process with less predictable outcomes. The co-roasting draws on the technical concept of growing apatite in molten salt (e.g., Calcium phosphide and Potassium Sulfate), thus excessive copper (or copper phosphate structures) should be a very important entity in apatite [17]. Apatite is generally regarded as a structurally stable phase; however, within this complex system, the interplay and equilibrium among multiple coexisting compounds are considered inevitable [18]. Consequently, the formation of phases such as ${\mathrm{Cu}}_{2}$S and insulating materials is an anticipated outcome of this research. Throughout the roasting process, the resultant products exhibit a strong correlation with the partial pressures of oxygen ($P_{O_{2}}$) and sulfur ($P_{S_{2}}$), indicating that apatite is not an unconditionally stable phase at elevated temperatures [19,20,21]. Under conditions of negligible sulfur partial pressure, an increase in oxygen partial pressure may facilitate further oxygen incorporation into the quasi-one-dimensional channels of the apatite structure, a concept supported by theoretical calculations in the existing literature [13,22]. Conversely, a reduction in oxygen partial pressure can lead to oxygen vacancies within these channels, potentially resulting in the formation of phosphates and a series of oxide impurities. At extremely low oxygen partial pressures, the formation of metallic copper and lead becomes thermodynamically favorable. The $P_{O_{2}}$-$P_{S_{2}}$ relationship is a fundamental consideration in copper–lead pyrometallurgy, where frequent substitution between sulfur and oxygen can ultimately lead to the generation of various products, including ${\mathrm{Cu}}_{2}$S and PbS [23,24]. Furthermore, substantial substitution of sulfur for oxygen within the apatite lattice is plausible, a phenomenon corroborated by studies on sulfur apatite.

In the apatite structure of lead phosphate, denoted as ${\mathrm{Pb}}_{10}{\left({\mathrm{PO}}_{4}\right)}_{6}O$, the oxygen ions at the Wyckoff 4e sites exhibit partial occupancy, specifically at a rate of 1/4 [25]. This inherent partial occupancy, a form of structural disorder, introduces a foundational complexity to the material’s crystal lattice. It has been identified in numerous theoretical models as a critical factor in modulating the system’s electronic properties. Although the claims of superconductivity in LK-99, a copper-doped variant of this apatite, have been conclusively refuted by a multitude of independent global experiments, the investigative process has been far from fruitless. On the contrary, these explorations have unveiled the copper-doped lead apatite system as a fertile platform for a rich tapestry of complex physical phenomena.

This report aims to move beyond a simple negation of the LK-99 superconductivity narrative. Instead, it seeks to delve into the new physics that has emerged from the intensive study of this material. We will systematically deconstruct the initial superconducting claims to build a new, more nuanced understanding of this material system. Our focus will be on exploring the intricate physical phenomena that are not mutually exclusive with superconductivity [26,27], such as those related to elemental doping, structural instabilities, strong electron–electron correlations, and defect chemistry.

## 2. Experimental Approach

### 2.1. Synthesis

**Figure 1:**
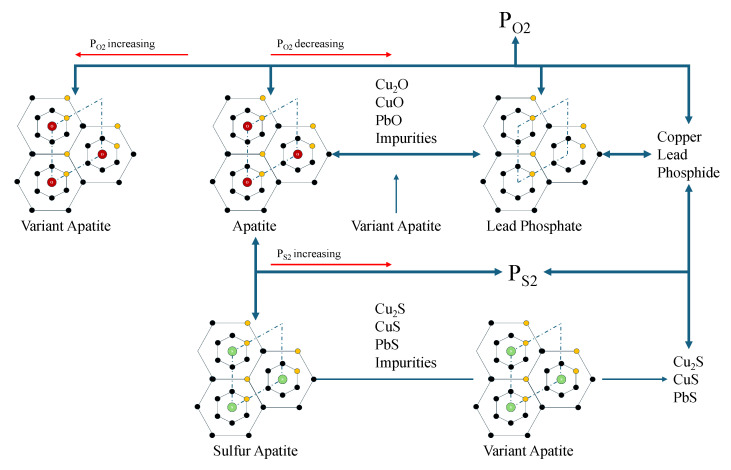
Schematic diagram illustrating the formation of apatite and associated impurity phases as a function of oxygen partial pressure ($P_{O_{2}}$) and sulfur partial pressure ($P_{S_{2}}$).

The schematic diagram in Figure 1 illustrates these potential reactions, offering deeper insight into the possible transformations occurring within the system. Therefore, it is crucial to acknowledge that the original experimental methodology inherently involves conditions of low oxygen potential and high sulfur partial pressure. The precise control of these parameters within a completely sealed system is challenging, leading to significant unpredictability in experimental outcomes. This lack of reproducibility does not contradict fundamental thermodynamic principles but rather highlights the complexities of controlling the reaction environment [28].

**Figure 2:**
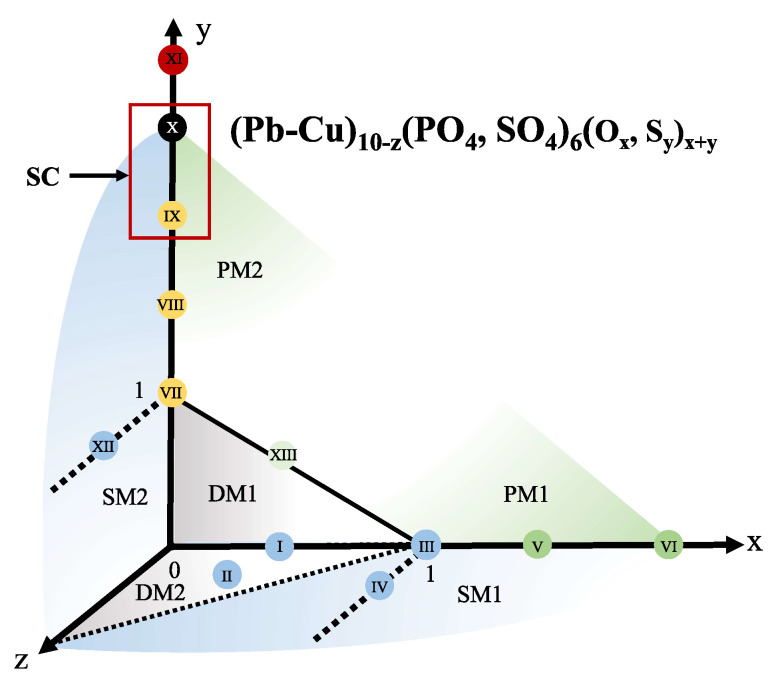
Synthetic phase diagram illustrates the preparation of samples labeled I-XIII. The general chemical formula for these samples is (Pb-Cu)_10−z_(${\mathrm{PO}}_{4}$, ${\left({\mathrm{SO}}_{4}\right)}_{6}$ $O_{x}$$S_{y}$. Pyrometallurgical synthesis primarily influences the oxygen atom count, *x*, while solvothermal methods predominantly alter the sulfur atom count, *y*. The quantity of metal ions, *z*, characterizes any unintentional transition from apatite to phosphate. The specific ratios of Pb/Cu are not considered significant in this context. We successfully obtained 13 samples exhibiting diverse magnetic phases, including diamagnetic (DM), paramagnetic (PM), soft magnetic (SM), and superconducting (SC) properties, with the numerical designations 1 and 2 indicating distinct origins of the magnetic moment.

The final products, synthesized via pyrometallurgy in oxygen or solvothermal methods in sulfide solutions with varying parameters, can generally be represented by the formula (Pb-Cu)_10−z_(${\left({\mathrm{PO}}_{4},\;{\mathrm{SO}}_{4}\right)}_{6}$$O_{x}$$S_{y}$ [29,30]. At this stage, precise determination of the copper and sulfate doping ratios is challenging and not critical. This is because various lead/copper and phosphate/sulfate feed ratios do not exhibit statistically significant differences in physical characteristics, partly due to the potential dissolution and decantation of copper ions from the raw materials. The fundamental synthetic parameters are the concentrations of oxygen and sulfur ions within the apatite channels [31,32], as illustrated in the synthetic phase diagram (Figure 2). Upon calcination in an oxygen-air atmosphere, *x* can be less than 1, indicating the presence of oxygen vacancies, or greater than 1, exceeding the normal ionic concentration within the apatite structure [33,34]. Further dispersion of lead–copper sulfoapatite in a sulfide solution can increase *y* to values even greater than 2 [35], resulting in the formation of persulfides. If sulfur doping is insufficient or the temperature is excessively high, *z* will not be eliminated, as lead may sublime, leading to the conversion of the product into lead phosphate, signifying a failed apatite synthesis. To elucidate the potential outcomes during the synthesis process, we prepared 13 distinct samples.

#### 2.1.1. ${\mathrm{Pb}}_{9}$${\mathrm{Cu}}_{1}$${\left({\mathrm{PO}}_{4}\right)}_{6}$$O_{x}$ Synthesis (I–VI)

The Samples I–IV, with a base structure of ${\mathrm{Pb}}_{9}$${\mathrm{Cu}}_{1}$${\left({\mathrm{PO}}_{4}\right)}_{6}$$O_{1\pm x}$, were all synthesized following a consistent procedure: co-precipitation, aging, hydrothermal treatment, and calcination. Throughout the aging and hydrothermal steps, a pH of 10 was maintained. The aging process occurred at 70°C, while the hydrothermal treatment was conducted at 150°C, with both steps lasting 24 h. For Samples II and IV, EDTA was additionally employed as a chelating agent during synthesis. This was done to simulate doping inhomogeneity by exploiting the differential binding affinities of EDTA with copper and lead [24,36]. Regarding calcination, Samples I and II were heated to 900°C, whereas Samples III and IV were subjected to a lower temperature of 500°C [37]. All calcination steps were carried out in air.

Sample V underwent a repeated low-temperature calcination process, followed by a 48 h heat treatment at 500°C in an oxygen atmosphere. This additional step aimed to increase the concentration of oxygen ions within the one-dimensional channels.

For Sample VI, during the co-precipitation stage, lead and copper hydroxides were substituted with their sulfide counterparts. This modification aimed to synthesize a chalcogenide apatite from the outset, ensuring the precise placement of copper ions. (Prior to this, DFT calculations were performed to determine the energy differences between various copper doping sites in both oxyapatite and sulfoapatite [38].) The subsequent calcination and oxygen heat treatment procedures for Sample VI mirrored those applied to Sample V.

#### 2.1.2. (Pb-Cu)_10_(${\mathrm{PO}}_{4}$, ${\left({\mathrm{SO}}_{4}\right)}_{6}$$S_{y}$ Synthesis (VII–X)

Sulfoapatite was synthesized via a hydrothermal method. In the initial hydrothermal stage, the molar ratio of Pb:Cu:P$O_{4}^{3-}$:$S^{2-}$ was controlled at (6–8):(2–4):6:2. A subsequent hydrothermal treatment involved the addition of supplementary $S^{2-}$ to the product from the first stage [39]. Both aging and hydrothermal processes were conducted following previously described methods, maintaining the pH between 7 and 8. The differing solubilities of PbS and CuS necessitate strict control over the reactant addition sequence and careful observation of product color during the hydrothermal process. For co-precipitation, the optimal reactant addition sequence was determined to be ${\mathrm{Cu}}^{2+}$, followed by ${\mathrm{PO}}_{4}^{3-}$, then ${\mathrm{Pb}}^{2+}$, and finally $S^{2-}$.

The characteristic color of sulfoapatite is black-gray; the appearance of other colors, such as blue, green, or brown, indicates an unsuccessful synthesis. Sample VII, designated sulfoapatite, was used as the precursor material for the second hydrothermal treatment and subsequent doping. The $S^{2-}$ concentration in the solution for the second hydrothermal stage, along with the duration of aging and hydrothermal treatment [35], directly influences the final product. The overall process involves the transformation from doped and distorted apatite to its eventual decomposition into sulfides.

Sample XII and Sample XIII represent two modifications of Sample VII. The former (XII) is a phosphate mixed-phase material obtained via a synthesis route that lacked a sufficient aging period. The latter (XIII) is the resultant product from the mild oxidation of Sample VII.

The synthesis details of the samples are summarized in Table 1. Considering the repeatability of sample synthesis, we will would the details and challenges involved in the synthesis process in Appendix A.

### 2.2. Characterization

The crystal structures of samples were analyzed using X-ray diffraction (XRD) (X’Pert PRO MPD, Malvern Panalytical B.V., Almelo, The Netherlands), operating at 40 kV and 25 mA with Cu-Kα radiation. The diffraction patterns were recorded over a 2θ range of 10°–90° with a step size of 0.02°. The micro-morphology and structure were analyzed using a field emission scanning electron microscope (SEM) (JSM-7800, JEOL, Tokyo, Japan) equipped with an energy-dispersive spectrometer (EDS) (Oxford Insturments, Oxford, UK). Additionally, transmission electron microscopy (TEM) (JEM-2100F, JEOL, Tokyo, Japan) was employed for further detailed observation and analysis of the microscopic morphology and structure. Detailed crystal parameters are shown in Appendix C Table A2.

The continuous-wave electron paramagnetic resonance (EPR) spectroscopy was measured on EPR spectrometer (Bruker ELEXSYS E580, Massachusetts, MA, USA) operating on the X-band (9.667 GHz) and outfitted with a dielectric resonator (ER-4118X-MD5, Bruker, Massachusetts, MA, USA). The microwave power was 15 dB, and the modulation amplitude was 5 Oe at 100 kHz. The magnetic field was corrected by using a BDPA standard (Bruker E3005313) with g=2.0026. Low-temperature environment was realized by an Oxford Instruments CF935 continuous-flow cryostat using liquid nitrogen. The temperature was controlled by an temperature controller (Oxford ITC4) with accuracy of ±0.1 K.

Based on SQUID detection technology, we conducted the dc magnetization measurement of the samples on MPMS-3. The holder is a capsule sample rod with almost no magnetism. Because the critical temperature of the samples is close to the room temperature, which makes them easily magnetized, even at low magnetic fields, it is necessary to demagnetize the sample at 380 K for 30 min before each testing cycle. The MH curves can only be measured by cooling the sample down from 380 K to eliminate the magnetization memory.

The electrical transport properties of Sample X were primarily investigated using a four-probe technique within a Physical Property Measurement System (PPMS) by Quantum Design (San Diego, CA, USA). A key consideration was the use of gold (Au) electrodes, chosen due to the sample’s potential persulfide nature, which could lead to sulfurization and compromised measurements if common materials like indium (In) or silver (Ag) were used. For resistance–temperature (R-T) characterization, samples were cooled to 2 K under zero magnetic field. Subsequently, the designated measurement current and magnetic field were applied, and resistance was recorded during a slow temperature increase (1 K intervals), with each data point averaged from 40 samplings. Complementary current–voltage (I–V) measurements were conducted using an Agilent B2912A Precision Source/Measure Unit (Santa Clara, CA, USA), with temperature control provided by an Oxford Instruments OptiStatDN cryostat. The Agilent unit was operated in “Compensating Resistance (R Compen)” mode, which automatically establishes an internal voltage offset on the inner probes before current application, meaning the initial measured voltage is non-zero even at zero applied current. A significant pre-measurement step involved applying a large current bias to the sample, a procedure intended to fill the defects. All I–V curves were averaged over 100 samplings to enhance data quality. The R-T and test details of Agilent test are shown in Appendix D, and the raw date photos during characterization was shown in Appendix F.

## 3. Discussion

### 3.1. Complex Magnetism and Possible Meissner Effect (I to VI)

**Figure 3:**
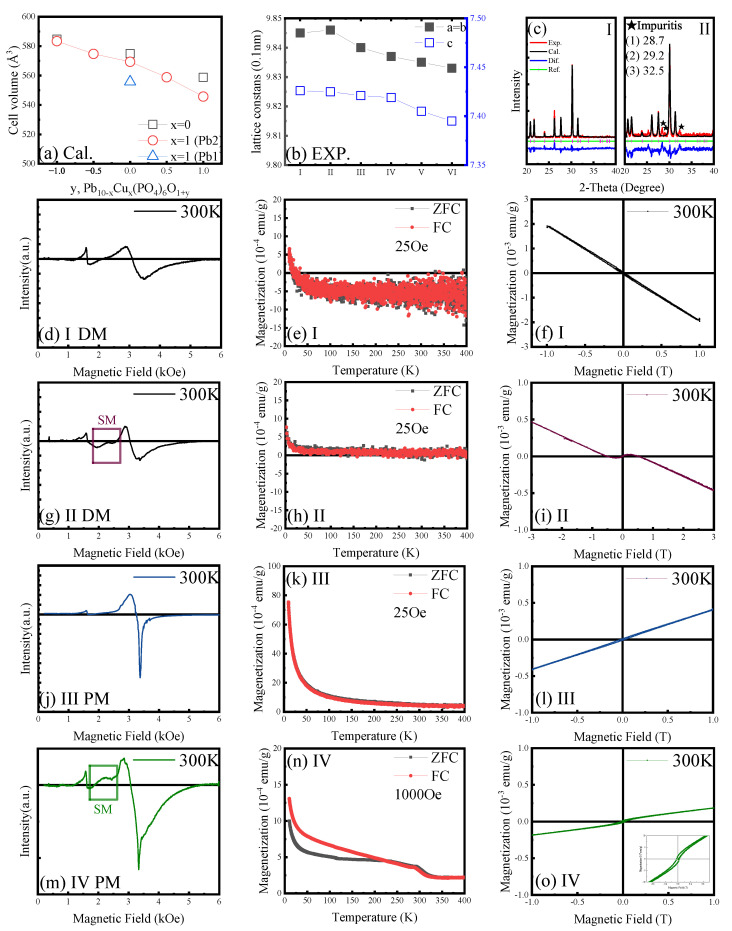
Complicated magnetism of Samples I–IV. (**a**) The calculated lattice constant as a function of atom number of oxygen in the 1D channels of lead apatite. An increase in oxygen concentration leads to an increase in lattice constant, which agrees with the realistic case that the lattice constant of lead phosphate with $P6_{3}/m$ space group is greater than that of lead apatite. (**b**) Lattice constants of Samples I to VI show a smooth decrease relationship with oxygen concentration increasing, in agreement with the calculations. (**c**) Comparison of XRD spectra of Samples I and II, in which the presence of three impurity peaks reveals the underlying mechanism of soft magnetism. (**d**–**o**) EPR spectra at 300 K, ZFC-FC MT curves and MH curves at 300 K of Samples I–IV, respectively. It is clear that the samples exhibit a transition from normal diamagnetism to paramagnetism. A weak soft magnetism can also be observed in Sample IV at low temperatures.

Figure 3 discussed the *x*-dependent influence on lattice and magnetic properties. Firstly, we calculated the *x*-dependent lattice constants of ${\mathrm{Pb}}_{9}$${\mathrm{Cu}}_{1}$${\left({\mathrm{PO}}_{4}\right)}_{6}$$O_{1\pm x}$. The optimal site for Cu substitution was determined to be the Pb(2) site [40] in the ${\mathrm{Pb}}_{9}$${\mathrm{Cu}}_{1}$${\left({\mathrm{PO}}_{4}\right)}_{6}$$O_{1\pm x}$ structure, which differs from the Pb(1) site as described for LK-99. The calculations are intended only for comparing the relative trends of change; the results indicate a slight contraction of the lattice constants with increasing *x*, which aligns with experimental observations.

Even in ambient air, a temperature of 900°C is sufficient to induce oxygen deficiency within the crystal. Conversely, oxygen annealing of Samples V and VI promoted lattice contraction. For the sample prepared with EDTA, some impurity peaks were detected. The observed soft magnetism is likely attributed to the non-uniform distribution of copper dopants. To mitigate potential interference from the sample holder during magnetic measurements, we calibrated the room-temperature magnetic properties using EPR. The signal observed in the range of 2000–2500 Oe can be interpreted as an indication of soft magnetism [41,42]. Consistent with experimental findings, the high temperature of 900°C promotes oxygen loss in the lead phosphate apatite structure [43], potentially leading to the formation of lead phosphate phases. In contrast, the sample calcined at 500°C exhibited paramagnetism. In the synthesized phase diagram, DM1 and DM2 are attributed to partial defects within the apatite structure of the samples, while SM1 is due to the non-uniform distribution of copper during synthesis. The precipitated copper oxides or copper-doped phosphates contribute to the observed soft magnetism.

**Figure 4:**
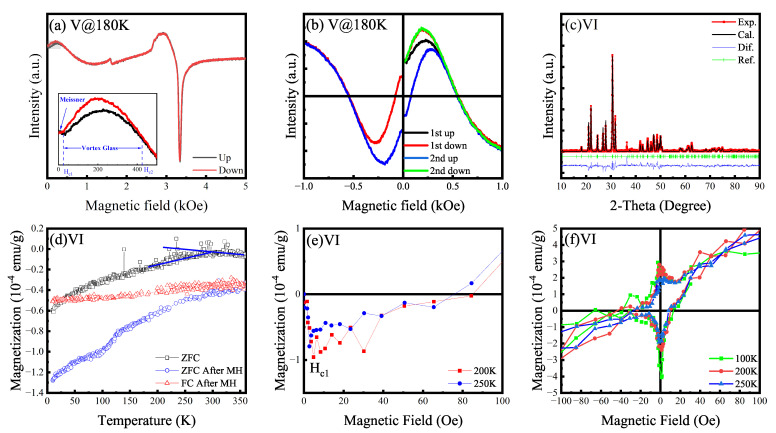
Possible Meissner effect in Samples V and VI. (**a**) EPR spectra of Sample V with the paramagnetism stemming from ${\mathrm{Cu}}^{2+}$. There is a low-field microwave absorption with observable hysteresis as displayed in inset of (**f**). (**b**) The hysteresis curves of low-field absorption in the first and second sweeps. The curves of negative field are obtained by rotating the sample by 180° and reversing the signs of both signal and magnetic field. (**c**) XRD pattern of Sample VI. (**d**) ZFC-FC MT curves exhibit diamagnetism below 270 K. Black curve represents the ZFC result after demagnetization at 380 K. Others hold magnetization memory after the MH measurement at 100 K. (**e**,**f**) show the initial magnetization curves and hysteresis loop of Sample VI provides a useful hint of superconductivity.

Sample V was synthesized by introducing additional oxygen atoms into the apatite framework of the paramagnetic Sample III. Sample V continued to exhibit strong paramagnetism as shown in Figure 4. EPR measurements, particularly with sample rotation, revealed a non-overlapping loop at low magnetic fields, indicative of hysteresis [44]. In the synthesis of Sample VI, sulfur was employed to stabilize copper (Cu) within the hydrothermally prepared precursor. It is posited that Cu preferentially substitutes at the Pb(2) crystallographic sites. Subsequent low-temperature sintering was designed such that a reduction in sintering temperature would not alter this site preference but would primarily facilitate S-O substitution and an excess incorporation of oxygen (over-doping). Sample VI exhibited pronounced diamagnetism, with a transition observed at approximately 270 K, indicative of a critical temperature ($T_{c}$). Furthermore, zero-field-cooled (ZFC) and field-cooled (FC) measurements were performed on Sample VI. Following an initial magnetization measurement at 100 K and subsequent return to zero field, the remeasured ZFC curve was observed to be lower than its pristine state. A distinct kink was also noted in the ZFC curve near 100 K, suggesting a glassy-state memory effect. Detailed measurements of the initial magnetization curve and the M-H hysteresis loop were conducted on Sample VI within a field range of ±100 Oe. The lower critical field ($H_{c1}$) was determined to be below 10 Oe, a parameter not extensively investigated in prior studies. At higher applied magnetic fields, the response of Sample VI was predominantly paramagnetic. Conversely, at fields below 100 Oe, a characteristic superconducting M-H hysteresis loop was clearly discernible. The signal-to-noise ratio of these measurements was relatively low, attributed to a small volume fraction of the superconducting phase within the sample. This hysteresis behavior was not detectable above 250 K. These findings for Sample VI bear some resemblance to the observations made for Sample V.

Consequently, the structural integrity of the apatite lattice appears to be a critical determinant distinguishing paramagnetic from diamagnetic behavior. Furthermore, excessive oxygen incorporation (over-doping) may potentially induce superconductivity, though quantifying the precise oxygen stoichiometry currently presents significant challenges. Additionally, observed diamagnetism could partially originate from lead phosphate phases, potentially formed via unintended decomposition during high-temperature roasting. Accurate identification of lead phosphate, particularly phases with the $P6_{3}/m$ space group, can be problematic in XRD analysis due to peak overlap or structural similarities. The observed complex magnetic behavior is experimentally linked to non-uniform substitution of copper for lead. This heterogeneity can, during calcination, lead to the formation of several undesired secondary phases, including copper oxides, copper phosphates, and copper-doped lead phosphates. Referencing the paramagnetic apatite in Sample III, the reported lattice constants for LK-99 are slightly larger. If the presence of a $P6_{3}/m$ lead phosphate phase is discounted, the data could suggest the possibility of a second superconducting phase existing under oxygen-deficient conditions. This hypothesis finds some support in the XPS data and reported synthesis conditions for LK-99 [4].

Furthermore, achieving uniform copper doping and precise oxygen stoichiometry control remains a significant challenge at present, and the emergence of soft magnetic behavior is plausible and difficult to entirely prevent, hindering the synthesis of a high-volume-fraction, homogeneously superconducting phase. It is posited that a structurally intact, paramagnetically responsive apatite phase with homogeneous copper doping serves as a crucial precursor for inducing superconductivity [45,46]. Nevertheless, the potential role of minor copper clustering in facilitating superconductivity upon further compositional modifications cannot be entirely dismissed and warrants more detailed experimental investigation [47,48]. Moreover, the pursuit of single-crystal synthesis [49] does not currently appear to be a viable pathway to obtaining the desired superconducting phase. Instead, resultant “jewels” may more closely resemble heterogeneous composites of lead phosphate and apatite, particularly if copper clustering occurs, rather than a bulk, single-phase superconductor.

### 3.2. Properties of Sulfoapatite (VII to IX)

**Figure 5:**
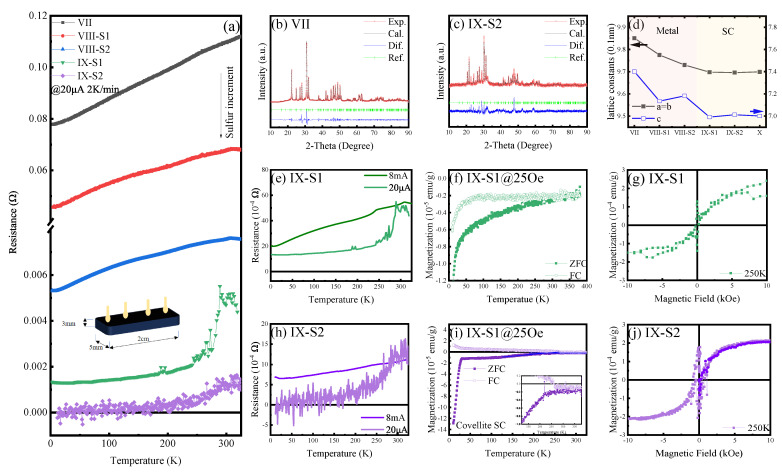
Physical properties of samples VII–IX by increasing the sulfur doping. (**a**) RT curves at 20 μA of five samples with S1 and S2 denoting parallel samples from the same synthetic procedure. Inset shows the measured samples have 2 cm in length, 5 mm in width, and 3 mm in thickness. (**b**,**c**) show XRD patterns of samples VII and IX-S2. Sample VII has a more complete apatite structure than that of IX-S2, which on the other hand manifests components of galena and covellite as indicated by the Dif. curve. (**d**) Calculated lattice constants of samples VII to X indicate substantial crystal shrinkage upon sulfur doping, leading to the transition from metal to superconductivity. (**e**) RT curves of Sample IX-S1 exhibit a remarkable jump at 20 μA and nearly linear change at 8 mA, indicating the large current breaks the zero-resistance state. (**f**) MT curves at 25 Oe of ZFC and FC measurements of Sample IX-S1. (**g**) Magnetic hysteresis loop at 250 K of Sample IX-S1. The parallel electric (**h**) and magnetic measurements (**i**,**j**) of Sample IX-S2. The low-temperature resistance is closer to zero than that of IX-S1.

Samples were fabricated into thin blocks (Figure 5a), with gold (Au) employed as the electrode material for four-probe resistivity measurements. Both Samples VII and VIII exhibited characteristic metallic behavior [50], indicated by a continuous change in resistance with temperature. In contrast, the heavily S-doped Sample IX demonstrated a sharp decrease in resistance upon temperature reduction. For comparative analysis, two parallel replicates of Sample IX, designated S1 and S2, were synthesized. The residual resistance was measured to be approximately 1 mΩ. The low-temperature resistance of S2 approached the detection limit of the Physical Property Measurement System (PPMS) and was therefore considered to be effectively zero.

XRD patterns for Samples VII and IX-S2 revealed that the primary phase was an apatite variant, exhibiting characteristic apatite peaks with slight crystallographic shifts; this phase was thus termed an “apatite variant.” For Sample IX-S2, additional diffraction peaks corresponding to covellite (CuS) and/or galena (PbS) were observed, indicating that excess sulfur formed these secondary phases with copper and lead, respectively. A distinct, abrupt change in lattice constants occurred subsequent to sulfur doping, signifying a structural transition that induced the transformation from a metallic to a superconducting state.

Resistance–temperature (R-T) characteristics were further investigated under high electrical current conditions. For Samples IX-S1 and S2, the sharp superconducting transition was suppressed, and the low-temperature resistance increased significantly. This observation indicates that high current densities disrupt the superconducting state, implying the existence of a critical current. Magnetization versus temperature (M-T) curves for both samples, acquired at 25 Oe, demonstrated superconducting diamagnetism during ZFC measurements, with a clear bifurcation between the ZFC and FC curves. At 250 K, both samples exhibited magnetic hysteresis loops below 1000 Oe. Collectively, these experimental findings provide compelling evidence that the samples transition into a superconducting phase above 250 K. Doping-induced structural distortions and sample thickness may contribute to non-uniform current distribution within the bulk material. Consequently, the phase transitions in Samples IX-S1 and S2, while qualitatively similar, may exhibit quantitative differences. From a thermodynamic equilibrium perspective, the formation of decomposition products concurrent with the structural distortion of apatite into its variant form is plausible.

### 3.3. Signature of Superconductivity in Sample X

**Figure 6:**
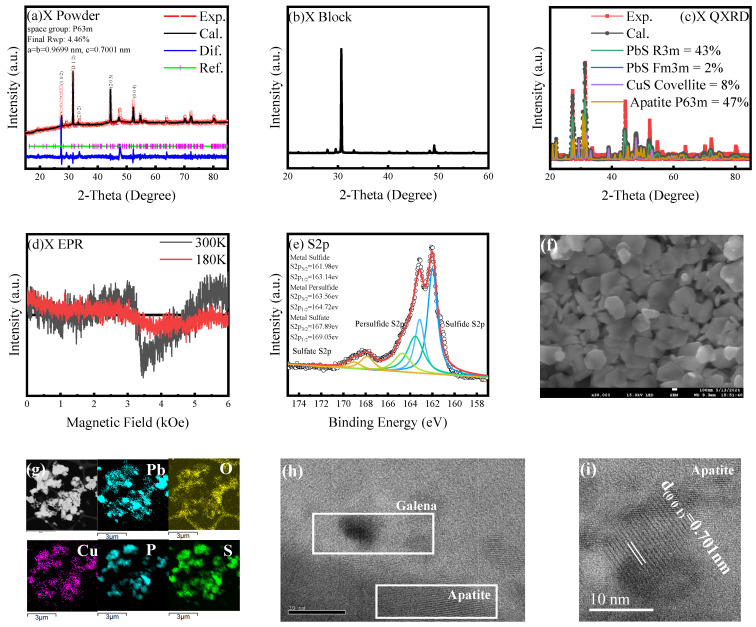
Structural characterization of Sample X. (**a**) XRD pattern of powdered Sample X is presented. The diffractogram exhibits distinct peaks corresponding to lead sulfide (PbS), indicating that the sample is a composite of apatite and PbS. (**b**) The XRD pattern of bulk Sample X reveals a singular, prominent peak, suggesting a significant degree of preferred orientation induced by applied pressure during sample preparation. (**c**) QXRD indicated that Sample X is mainly a blend of apatite and galena. (**d**) EPR spectra of Sample X demonstrate paramagnetic behavior at 300 K, which is absent at 180 K. (**e**) XPS data for the S 2p orbital, upon deconvolution, reveal three distinct chemical states, qualitatively assigned to sulfide, persulfide, and sulfate species. The substitution of oxygen by sulfur in the apatite channel may concurrently lead to the replacement of ${\mathrm{PO}}_{4}^{3-}$ groups. This inherent uncertainty contributes to challenges in experimental reproducibility. (**f**) displays morphology of Sample X. (**g**) Higher magnification imaging further elucidates the sample’s morphology, accompanied by an energy-dispersive X-ray spectroscopy (EDS) map. The EDS analysis confirms the homogeneous distribution of lead (Pb), phosphorus (P), sulfur (S), and oxygen (O) within the product particles, indicating the coexistence of apatite (phosphate) and sulfide phases. (**h**) A TEM image of Sample X, with lattice fringe analysis, reveals the structural distribution and interrelationship between galena (PbS) and apatite phases. (**i**) Further analysis of the lattice fringes in the TEM image allowed for the measurement of the (001) interplanar spacing of apatite, determined to be 7.01 Å. This value is in close agreement with the results obtained from Rietveld refinement of the XRD data.

To elucidate complex material phenomena, a novel composite, designated Sample X, was synthesized and subjected to comprehensive material characterization. XRD analysis formed the initial basis of structural investigation, as depicted in Figure 6. The XRD pattern of Sample X unequivocally revealed a biphasic composition, exhibiting two distinct sets of diffraction peaks. One set was identified as corresponding to a variant apatite phase, and the second set of peaks was unambiguously assigned to galena. A notable feature in the bulk XRD pattern of Sample X was the evidence of preferential grain orientation. This phenomenon is frequently attributed to directional stacking or alignment of crystallites during the high-pressure compaction stage of material synthesis, which can induce texture in the sample. Furthermore, the galena phase, which crystallizes in the face-centered cubic Fm$\bar{3}$m space group, presented an interesting crystallographic feature. Achieving controlled in-plane growth of the (111) crystal plane of galena is typically a significant crystallographic challenge, suggesting specific growth dynamics in this composite system [51]. Morphological investigations using nanosheet imaging techniques provided further insights into the microstructural organization of Sample X. The observation of a distinct hexagonal morphology in the imaged nanosheets suggests an intimate association or potential intergrowth between the variant apatite and galena phases. This microstructural arrangement is hypothesized to originate during the hydrothermal synthesis process. It is proposed that PbS, formed from the decomposition of precursors under hydrothermal conditions, undergoes epitaxial growth on the surface of the distorted variant apatite. Epitaxy would facilitate the formation of a coherent “Variant apatite@PbS” core-shell or intergrown structure, consistent with the observed hexagonal morphology.

Transmission electron microscopy (TEM) analysis provided high-resolution structural data. Specifically, the interplanar spacing of the (001) crystallographic plane for the variant apatite phase was determined to be 7.01 Å. This experimental value is in strong agreement with data obtained from the refinement of the X-ray diffraction patterns, lending confidence to the structural parameters derived for the apatite phase. To probe the electronic properties, particularly in relation to potential superconductivity, electron paramagnetic resonance (EPR) spectroscopy was employed. EPR spectra, recorded at room temperature (300 K) and a lower temperature of 180 K, revealed a critical change. A weak paramagnetic signal, indicative of unpaired electron spins present at 300 K, was observed to disappear upon cooling to 180 K. The quenching of this paramagnetic signal at reduced temperatures is a significant observation. In many material systems, such a disappearance is considered strong evidence for a transition to a superconducting state. This is because, in the superconducting state, electrons form Cooper pairs (which are spinless and EPR silent) and/or the Meissner effect expels the magnetic field essential for EPR resonance [52].

Further chemical state analysis was conducted using X-ray Photoelectron Spectroscopy (XPS). The XPS results provided evidence suggesting the potential presence of sulfate (${\mathrm{SO}}_{4}^{2-}$) groups within Sample X [53,54]. This finding has important implications for the material’s chemistry and structure. The presence of such sulfur species may indicate an increased sulfur capacity [55], potentially accommodated within quasi-one-dimensional channels known to exist in some apatite structures. Moreover, this observation, coupled with the known presence of copper, suggests the possible formation of a continuous chain structure involving copper ions, which could be crucial for the observed electronic properties.

**Figure 7:**
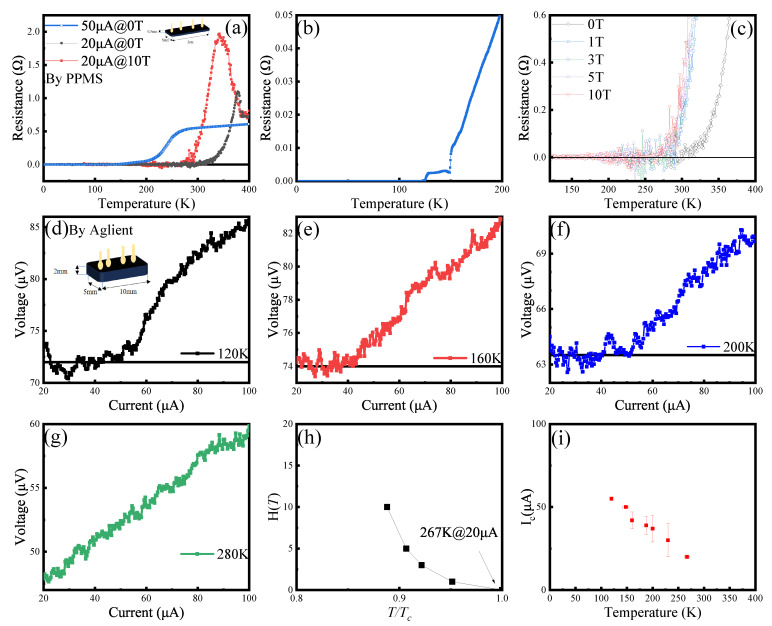
Electrical transport properties. (**a**) Resistance versus temperature (R-T) curves measured under various applied magnetic fields using a PPMS are presented. An abrupt transition to zero resistance is consistently observed. The critical temperature ($T_{c}$) decreases with increasing magnetic field strength and is determined to be in the range of 265–270 K at an applied current of 20 μA. (**b**) Detailed R-T data acquired at an applied current of 50 μA are shown. Upon increasing the current, the resistive transition temperature shifts to a range between 120 K and 130 K. (**c**) The details of the resistive transition under different magnetic fields are illustrated, revealing significant fluctuations in the measured resistance near the transition point. (**d**–**g**) Current–voltage (I–V) characteristics measured at four distinct temperatures using an Agilent system are displayed. The critical current ($I_{c}$) at 120 K is approximately 55 μA, while at 160 K, it is less than 40 μA. These findings are consistent with the R-T measurement results. (**h**) The relationship between the critical temperature ($T_{c}$) and the applied magnetic field is depicted for measurements conducted at 20 μA. The critical temperature, $T_{c}$, was determined from the derivative of the resistance with respect to temperature ($\frac{dR}{dT}$) due to the broadness of the transition. The derivative curve was smoothed using an adjacent-averaging method to minimize noise. (**i**) The relationship of temperature and $I_{c}$. With a lower test limit of 20 μA, we are hopeful that a reduced current will enable us to identify the I-V plateau at higher temperatures.

Resistance–temperature (R-T) curves for Sample X provided clear evidence of high-temperature superconductivity. With an applied current of 20 μA, the sample exhibited a superconducting transition temperature ($T_{c}$) in the range of 265 K to 270 K (Figure 7). This transition was described as a “smooth progression.” Further supporting the superconducting nature, an increase in applied magnetic field led to a systematic decrease in $T_{c}$, a hallmark characteristic of superconductors. In the normal state, above $T_{c}$, Sample X displayed anomalous resistance behavior. As temperature increased beyond $T_{c}$, resistance first rose sharply and then subsequently decreased. This non-monotonic trend was attributed to the destruction of superconducting coherence and the disruption of interference between transport channels [56]. This behavior was noted as being commonly observed in high-temperature superconductors, suggesting complex electronic phenomena, possibly related to strong electronic correlations [57,58]. Sample X demonstrated a pronounced sensitivity of its $T_{c}$ to the applied electrical current; increasing the current to 50 μA suppressed the $T_{c}$ to below 150 K. Resistance fluctuations near $T_{c}$ under a 20 μA current were attributed to non-uniform current distribution within the sample, a phenomenon described as common in superconductor testing.

Direct current–voltage (I–V) characteristics confirmed the zero-resistance state. This plateau, where voltage remains effectively zero up to a critical current, was completely absent at 280 K, indicating the sample was in its normal, resistive state. The critical current ($I_{c}$) showed temperature dependence, decreasing from over 50 μA at 120 K to 40 μA at 160 K. This implies that for currents exceeding 40 μA, the sample is not superconducting at 160 K. The findings from these I–V measurements were highly consistent with the R-T curve, providing strong cross-validation.

Electrical resistivity measurements on Sample X revealed a distinct transition where the resistance decreased significantly. While a drop in resistance is a necessary indicator of a superconducting transition, it is not, in isolation, sufficient proof. The ideal single crystal sample was not successfully synthesized; according to the structural analysis mentioned above, the observation of zero resistance is more likely relied on the proximity effect [59,60]. Such transitions can be mimicked by other physical phenomena, such as metal–metal phase transitions or even artifacts from inhomogeneous current distribution in multiphase samples. The electrical measurements were conducted using a low current of 20 μA, which is a prudent experimental choice to minimize potential issues like sample self-heating or current-induced suppression of a fragile superconducting state, especially if the critical current ($I_{c}$) is low. Nevertheless, achieving and confirming a true zero-resistance state is experimentally challenging, and without corroborating magnetic data, a sharp resistance drop remains ambiguous.

**Figure 8:**
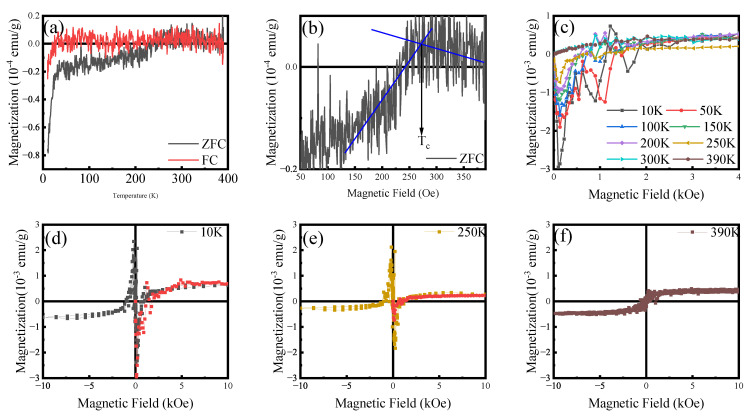
Magnetic properties. (**a**) ZFC-FC MT curves at magnetic field of 25 Oe. A transition from paramagnetism to diamagnetism occurs at around 270 K, and below 30 K, the magnetization dramatically drops. (**b**) The details of ZFC show a transition temperature of approximately 270 K. (**c**) Initial magnetization curves at eight temperatures. (**d**–**f**) MH curves at 10 K, 250 K, and 390 K, with linear diamagnetic background subtracted. The former two exhibit superconducting hysteresis, and the latter one is soft magnetism.

The DC magnetization of Sample X was investigated using a SQUID magnetometer, with the results presented in Figure 8. ZFC and FC magnetization curves both exhibit a distinct diamagnetic signal below 270 K, which is characteristic of superconductivity. This observation is consistent with electrical transport measurements conducted at 20 μA, which identify this temperature as the critical temperature ($T_{c}$). Two distinct superconducting phases are evident: The first phase, observed in the range of 30 K to 270 K, can be attributed to a near-room-temperature superconducting phase associated with a variant apatite structure. The second phase, emerging below 30 K, is considered to be a low-temperature superconducting phase predominantly originating from a sulfide component [61]. The inherent diamagnetic background is subtracted in the M-H date processing, and the specific process is supplemented in Appendix E. Initial magnetization curves and magnetic hysteresis loops were measured at various temperatures. The observed hysteresis loops are characteristic of superconductors [62,63]. All superconducting signatures are clearly discernible and consistent with other experimental results. Notably, in its normal state, the sample exhibits weak soft magnetic behavior [64], which is associated with the morphology resulting from copper doping and nano-particle [65]. However, the superconducting phase proportion in Sample X remains very weak, and the order of magnitude of its magnetic susceptibility has been a subject of significant controversy in past research.

In the case of ordinary diamagnetism, electronic orbitals generate induced currents that oppose the external magnetic field according to Lenz’s law, and the net magnetic moment exhibits a linear dependence on the field with a negative slope. By contrast, superconducting diamagnetism weakens with increasing magnetic field because the applied field progressively disrupts the superconducting state, leading to flux penetration or even the suppression of the superconducting phase. The “false” diamagnetism, such as that of copper or lead, falls into the category of ordinary diamagnetism and strictly follows the linear negative-slope relation with the magnetic field. False diamagnetism cannot produce sharp peaks near zero field, as observed in Figure 4, Figure 5 and Figure 8, which are signatures uniquely detectable in superconductors. Nonetheless, we still need to synthesize higher-purity samples to provide more substantial evidence.

### 3.4. Distortion and Decomposition Process (XI to XIII)

**Figure 9:**
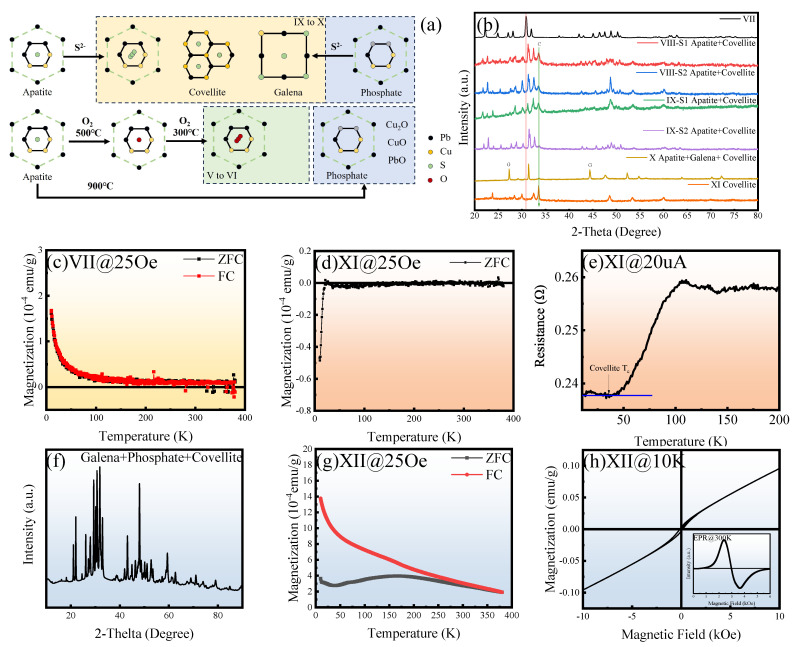
Synthesis nodes and extreme cases of variant apatite. (**a**) Schematic diagram illustrating the synthesis nodes and mixed states of variant apatite. (**b**) X-ray diffraction (XRD) patterns of samples at different synthetic stages, indicating peak position shifts. (**c**) Magnetic susceptibility data for Sample VII, representing the onset of deep sulfur doping, demonstrating paramagnetic behavior. (**d**) Zero-field-cooled (ZFC) curve for a sample after decomposition into covellite, independently indicating a potential low-temperature superconducting phase. (**e**) Corresponding temperature-dependent electrical resistance curve for the sample in (**d**); due to the mixed-phase nature of the sample, the resistance does not strictly reach zero, yet the transition is clearly discernible. (**f**) XRD pattern of an extreme ferromagnetic sample (XII), where insufficient aging precluded the formation of apatite and the initial framework. (**g**,**h**) Magnetic characteristics of Sample VII, with panel (**h**) including an inset displaying its electron paramagnetic resonance (EPR) spectrum. Objectively, EPR serves as an efficient tool for rapidly screening a large number of samples to identify those that do not warrant in-depth investigation.

This study necessitates a thorough examination of potential issues arising during synthesis and the composition of complex mixtures. As previously discussed, variant apatite is identified as an intermediate product in the decomposition of apatite to sulfides under high $P_{S_{2}}$ (sulfur partial pressure) or concentrated $S^{2-}$ ion solutions. Its structure is inherently unstable, existing within a multiphase equilibrium. As illustrated in Figure 9, the doping process can be delineated into several intermediate phases, each characterized by the predominant active components within the mixture: the initial apatite raw material, variant apatite, and a mixture of variant apatite with covellite and galena [66,67]. Sulfur doping concurrently induces structural distortion and a reduction in lattice constants, consequently leading to a significant decrease in electrical resistance. When the doping level surpasses a critical threshold, exceeding the accommodation capacity of the one-dimensional channels, the apatite structure will completely decompose into covellite and galena. Furthermore, insufficient sulfur doping in the initial stage or excessively high temperatures during the sintering process will cause the apatite to decompose into phosphate, thereby rendering any subsequent doping attempts futile.

XRD patterns reveal that lightly doped samples retain the pristine apatite framework. However, with increasing doping levels, the apatite structure evolves and approaches a state of collapse, facilitating the formation of sulfides and polysulfides. Figure 9c–e depict crucial junctures in the magnetic evolution, specifically the paramagnetic behavior of Sample VII and the low-temperature superconductivity of Sample XI. The electrical resistance of Sample XI, predominantly composed of covellite, exhibits a sharp decline around 30 K, consistent with the magnetization temperature. This observation further substantiates the low-temperature superconducting properties of covellite.

It is pertinent to note that the differentiation between apatite and $P6_{3}/m$ phosphate via XRD is challenging due to their similar space groups. Consequently, through analogous synthetic procedures, there exists a potential to synthesize samples, such as XII, that are primarily composed of phosphate. The distinguishing factor in such syntheses is an insufficient aging process. The potential presence of a ferromagnetic phase [19] within this system can be effectively identified by both magnetic measurements and electron paramagnetic resonance (EPR), a finding that enriches the phase assemblage of apatite/phosphate materials [68].

**Figure 10:**
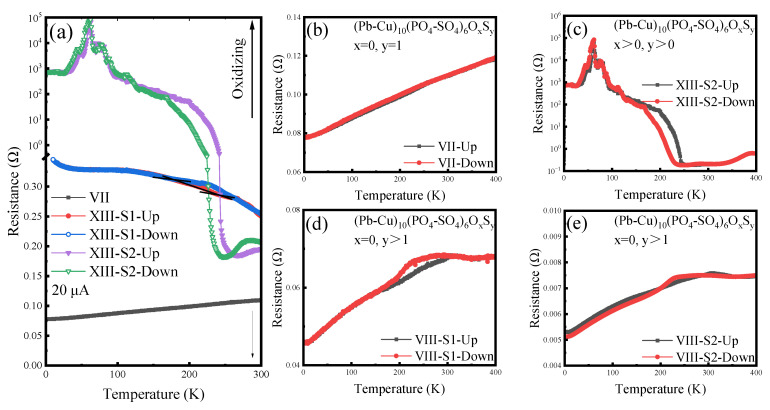
Electrical transport properties of semiconducting samples. (**a**) Resistance versus temperature (R-T) curves for samples VII and XIII. Sample XIII, subjected to oxidation in air, exhibits semiconducting characteristics alongside an unexpected phase transition between 200 K and 300 K, potentially indicative of strong correlation effects. (**b**) Sample VII displays typical metallic conductivity. (**c**) Logarithmic plot of the R-T curve for Sample XIII-S2, illustrating metallic behavior at room temperature transitioning to semiconducting behavior at approximately 240 K. (**d**,**e**) R-T curves for parallel samples VIII-S1 and VIII-S2, respectively. With moderate sulfur doping, these samples exhibit deviations from standard metallic conductivity.

Subsequently, Sample VII was subjected to controlled oxidation in air at 300°C, resulting in the emergence of semiconducting properties (Figure 10). This suggests that the unstable variant apatite may have undergone decomposition and/or S-O (sulfur–oxygen) substitution. Significant phase transitions were still observable in these oxidized samples. Notably, Sample XIII exhibited a distinct metal–insulator transition, characterized by an exponential increase in resistance upon temperature reduction. The doping differences lead to significant changes in physical properties [69,70]. This novel physical phenomenon had not been observed in the previous replication of LK-99’s experiments [71]. For completeness, we also present the temperature-dependent resistance curve for Sample VIII. With continued sulfur doping, a clear phase transition feature emerged in the 180 K to 300 K range. Considering the complex nature of the samples, this behavior could be attributed either to a unique structural configuration of apatite or to a dispersed weak link between superconducting and semiconducting (or metallic) phases [72].

## 4. Conclusions

This research comprehensively investigated the complex physical properties of the Pb-Cu-P-S-O apatite system, revealing a remarkable diversity of behaviors—a “material phase zoo”—and presenting compelling signatures of possible near-room-temperature superconductivity. The study systematically explored the effects of varying doping ratios of sulfur and oxygen, alongside different synthesis conditions, leading to materials that span semiconducting, metallic, and potentially superconducting states, each exhibiting unique magnetic characteristics. While the observed diamagnetic transitions and sharp resistance drops, particularly in Samples VI, IX, and X, strongly suggest superconductivity above 250 K, we acknowledge the challenges in reproducibility, precise stoichiometric control, and the low-volume fraction of the superconducting phase in heterogeneous samples. The “variant apatite” structure, heavily doped with sulfur, appears crucial but is also prone to decomposition. Future efforts must focus on achieving higher-purity samples, refining synthesis protocols for better control over doping and impurity suppression. Despite the existing hurdles, the findings presented offer a case for apatite as a promising, albeit complex, platform for high-temperature superconductivity research and contribute valuable insights into the intricate interplay of structure, doping, and emergent physical properties in these materials.

## Figures and Tables

**Table 1:** Synthesis parameters for lead–copper apatite derivatives.

Target: ${\mathrm{Pb}}_{9}$${\mathrm{Cu}}_{1}$${\left({\mathrm{SO}}_{4}\right)}_{6}$$O_{x}$						
Order	Step 1	Product 1	Step 2	Temp. (°C)	Chelating Agent	Atmosphere
I	H	A	R	900	EDTA	Air
II	H	A	R	900	EDTA	Air
III	H	A	R	500	—	Air
IV	H	A	R	500	—	Air
V	H	A	R	500	—	Air
VI	H	B	R	500	—	$O_{2}$
**Target: (Pb,Cu)_10_(${\mathrm{PO}}_{4}$, ${{\mathrm{SO}}_{4})}_{6}$$S_{y}$**						
**Order**	**Step 1**	**Product 1**	**Step 2**	**SIC (mol/L)**	**Period (h)**	**pH (@ 25 °C)**
VII	H	C	—	—	—	7–8
VIII	H	C	H	0.1	24	7–8
IX	H	C	H	0.2	24	7–8
X	H	C	H	0.2	24	7–8
XI	H	C	H	0.3	24	7–8
**Target: (Pb,Cu)_10−*z*_ (${\mathrm{PO}}_{4}$, ${{\mathrm{SO}}_{4})}_{6}$$O_{x}S_{y}$**						
**Order**	**Step 1**	**Product 1**	**Step 2**	**Temp. (°C)**	**Period (h)**	**Atmosphere**
XII	H	D	—	—	—	—
XIII	H	C	R	300	3	Air

H: Hydrothermal; R: Roasting; SIC: Sulfur ion Conc.; A: ${\mathrm{Pb}}_{9}$${\mathrm{Cu}}_{1}$${\left({\mathrm{SO}}_{4}\right)}_{6}$(OH)_2_; B: ${\mathrm{Pb}}_{9}$${\mathrm{Cu}}_{1}$${\left({\mathrm{SO}}_{4}\right)}_{6}S$; C: (Pb,Cu)_10_ ${\left({\mathrm{SO}}_{4}\right)}_{6}S$; D: (Pb,Cu)_10−z_ ${\left({\mathrm{SO}}_{4}\right)}_{6}S$.

**Figure A1:**
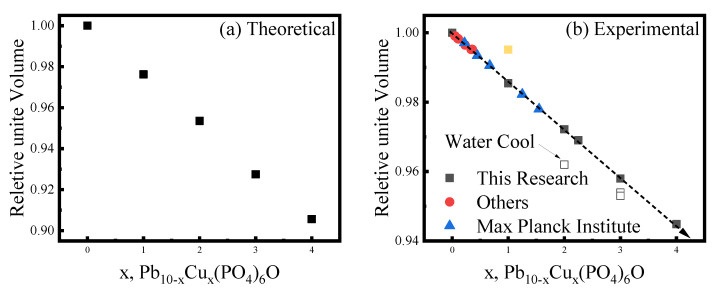
Variation of the relative unit cell volume with different Cu doping levels. (**a**) Theoretically calculated values by DFT, where all copper doping sites were assumed to be (Pb2). (**b**) Experimental results from the present study with a linear regression analysis. Data from other researchers uniformly indicate a lower-than-expected level of Cu doping. The orange point marked the data corresponding to LK-99.

**Figure A2:**
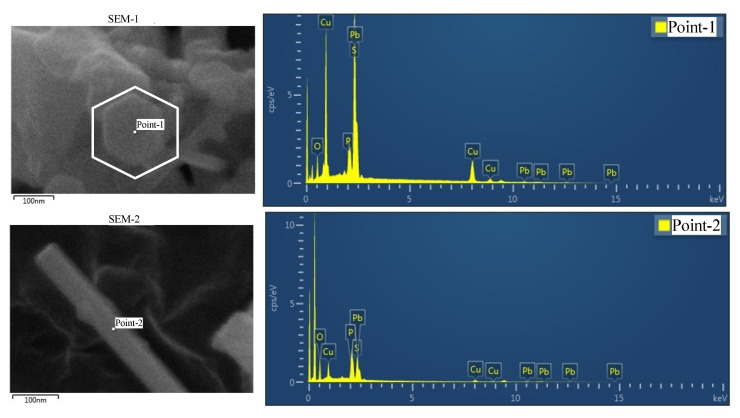
SEM-EDS data of apatite and sulfide blends.

**Figure A3:**
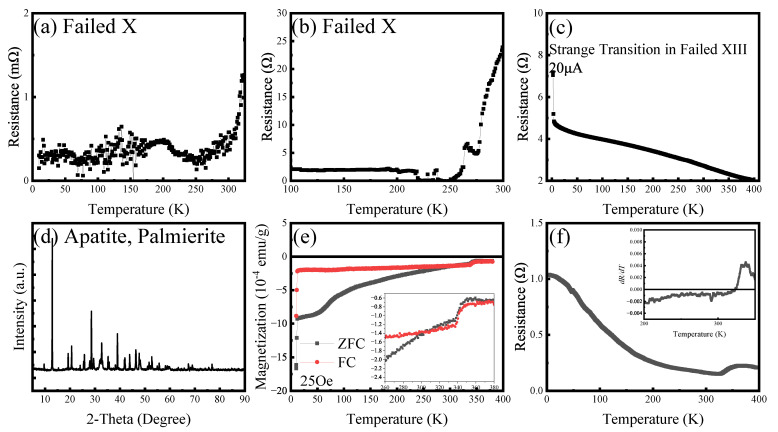
Properties of replicated samples. (**a**,**b**) illustrate our replication studies on Sample X. While a distinct resistive transition was consistently identified above 250 K, achieving true zero resistance proved difficult to replicate due to issues concerning sample quality by ourselves. This could be attributed to the sample’s powder nature. (**c**) shows data from the replicated Sample XIII. Macroscopically, the sample demonstrates semiconducting behavior, yet a significant change in resistance is observed at temperatures below 4 K. (**d**) illustrates a potential composition for the Pb-Cu-P-O-S compound. The corresponding zero-field-cooled (ZFC) and field-cooled (FC) magnetization curve and resistance–temperature (R-T) curve are depicted in Figure (**e**,**f**). The magnetic transition occurs above 320 K, consistent with the electrical resistance data. The presence of some residual impurities, which could not be eliminated, contributes to a background semiconducting resistance, despite the observation of a clear phase transition.

**Figure A4:**
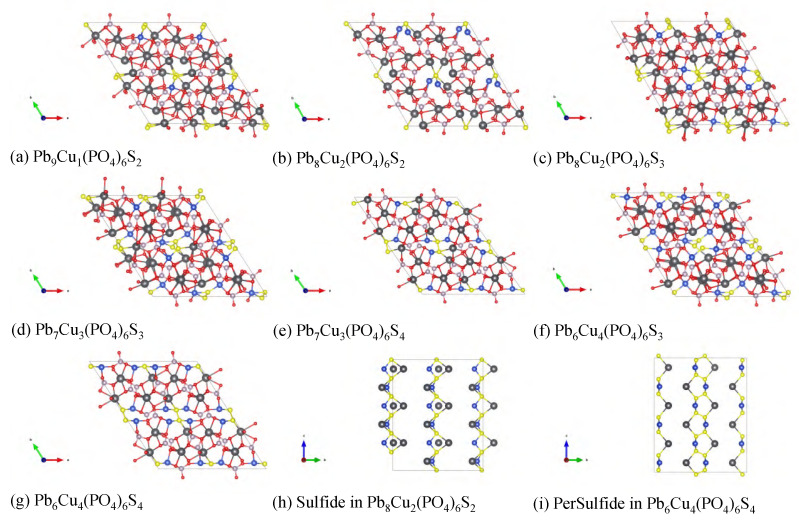
Optimal variant–apatite structure (**a**) ${\mathrm{Pb}}_{9}$Cu${\left({\mathrm{PO}}_{4}\right)}_{6}$$S_{2}$, (**b**) ${\mathrm{Pb}}_{8}$${\mathrm{Cu}}_{2}$${\left({\mathrm{PO}}_{4}\right)}_{6}$$S_{2}$, (**c**) ${\mathrm{Pb}}_{8}$${\mathrm{Cu}}_{2}$${\left({\mathrm{PO}}_{4}\right)}_{5}$$S_{3}$, (**d**) ${\mathrm{Pb}}_{7}$${\mathrm{Cu}}_{3}$${\left({\mathrm{PO}}_{4}\right)}_{6}$$S_{3}$, (**e**) ${\mathrm{Pb}}_{7}$${\mathrm{Cu}}_{3}$${\left({\mathrm{PO}}_{4}\right)}_{6}$$S_{4}$, (**f**) ${\mathrm{Pb}}_{6}$${\mathrm{Cu}}_{4}$${\left({\mathrm{PO}}_{4}\right)}_{6}$$S_{3}$, (**g**) ${\mathrm{Pb}}_{6}$${\mathrm{Cu}}_{4}$${\left({\mathrm{PO}}_{4}\right)}_{6}$$S_{4}$, and (**h**) quasi-one-dimensional in ${\mathrm{Pb}}_{9}$Cu${\left({\mathrm{PO}}_{4}\right)}_{6}$$S_{2}$ and (**i**) ${\mathrm{Pb}}_{6}$${\mathrm{Cu}}_{4}$${\left({\mathrm{PO}}_{4}\right)}_{6}$$S_{4}$.

**Figure A5:**
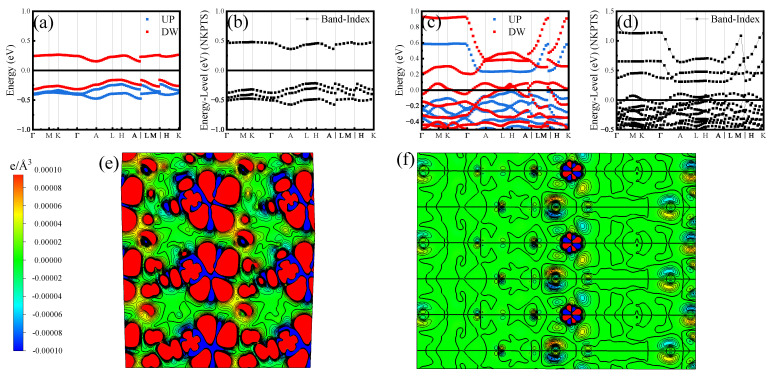
(**a**) Band structure of ${\mathrm{Pb}}_{9}$Cu${\left({\mathrm{PO}}_{4}\right)}_{6}$$S_{2}$, and (**b**) the spin-orbit coupling. (**c**) Band structure of ${\mathrm{Pb}}_{8}$${\mathrm{Cu}}_{2}$${\left({\mathrm{PO}}_{4}\right)}_{6}$$S_{2}$, and (**d**) the spin-orbit coupling. (**e**) Two-dimensional magnetic map in ${\mathrm{Pb}}_{9}$Cu${\left({\mathrm{PO}}_{4}\right)}_{6}$$S_{2}$, and (**f**) ${\mathrm{Pb}}_{8}$${\mathrm{Cu}}_{2}$${\left({\mathrm{PO}}_{4}\right)}_{6}$$S_{2}$.

**Figure A6:**
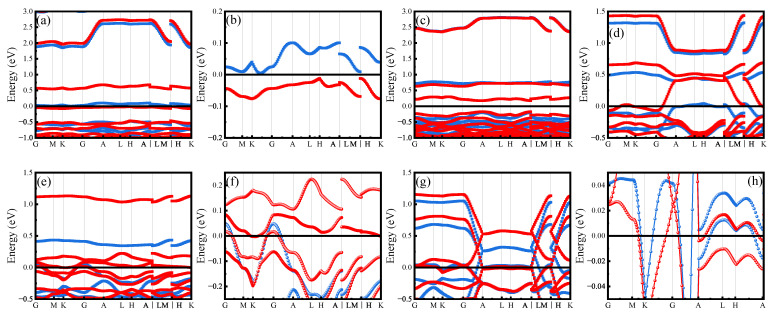
Band structure of (**a**,**b**) ${\mathrm{Pb}}_{8}$${\mathrm{Cu}}_{2}$${\left({\mathrm{PO}}_{4}\right)}_{5}$$S_{3}$, (**c**) ${\mathrm{Pb}}_{7}$${\mathrm{Cu}}_{3}$${\left({\mathrm{PO}}_{4}\right)}_{6}$$S_{3}$, (**d**) ${\mathrm{Pb}}_{7}$${\mathrm{Cu}}_{3}$${\left({\mathrm{PO}}_{4}\right)}_{6}$$S_{4}$, (**e**,**f**) ${\mathrm{Pb}}_{6}$${\mathrm{Cu}}_{4}$${\left({\mathrm{PO}}_{4}\right)}_{6}$$S_{3}$, and (**g**,**h**) ${\mathrm{Pb}}_{6}$${\mathrm{Cu}}_{4}$${\left({\mathrm{PO}}_{4}\right)}_{6}$$S_{4}$.

**Figure A7:**
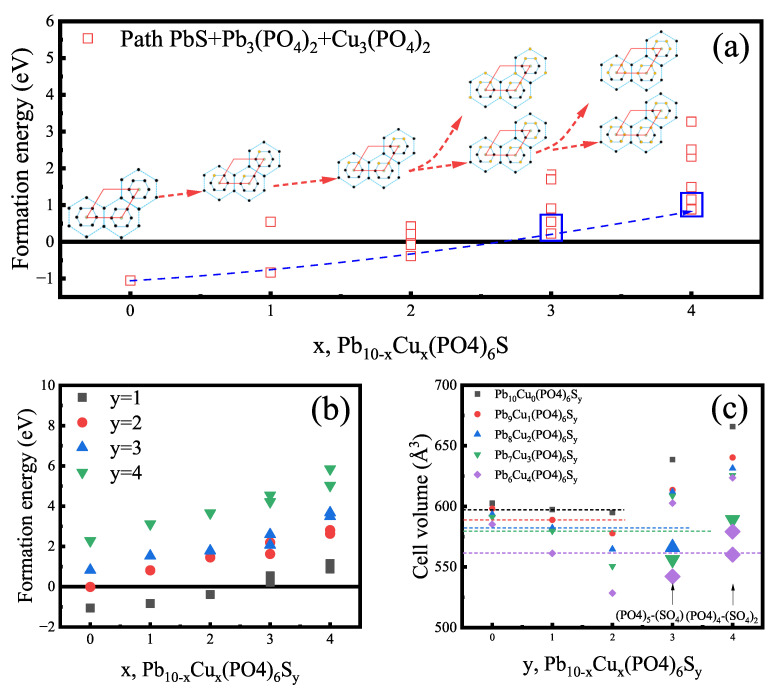
Theoretical calculation results. (**a**) Formation energies for different Cu doping sites. The reference path for these calculations was a mixture of galena and phosphate, with structural optimization and energy calculations performed independently for the initial raw materials. (**b**) The formation energies for various copper and sulfur doping concentrations in the optimized configuration are presented. (**c**) The effect of different sulfur doping levels on the unit cell volume is shown. Additionally, we have supplemented this with an analysis of the potential impact on the lattice parameters resulting from the substitution between sulfate (${\mathrm{SO}}_{4}^{2-}$) and phosphate (${\mathrm{PO}}_{4}^{3-}$) groups.

**Figure A8:**
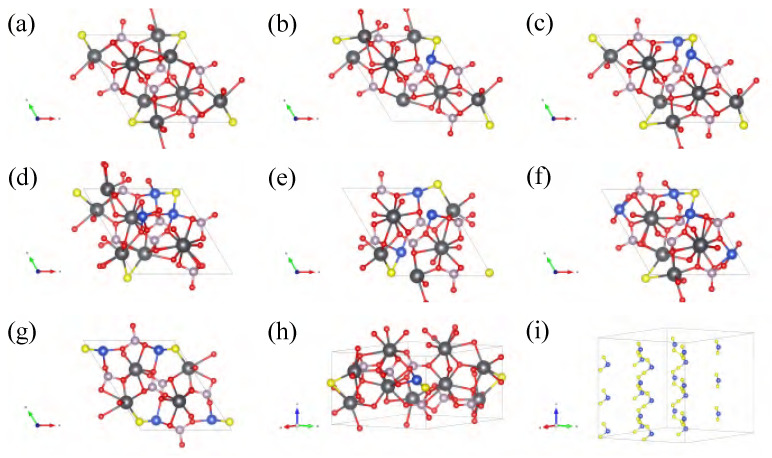
Crystal structures of ${\mathrm{Pb}}_{10-x}$${\mathrm{Cu}}_{x}$${\left({\mathrm{PO}}_{4}\right)}_{6}$$S_{y}$ for (**a**) x=0,y=1,2,3,4, (**b**) x=1,y=1,2,3,4, (**c**) x=2,y=1,2,3,4, (**d**) x=3,y=1, (**e**) x=3,y=2,3,4, (**f**) x=4,y=1, and (**g**) x=4,y=2,3,4. (**h**) Detailed structure for x=1,y=1. (**i**) The Cu-S polymer chain in ${\mathrm{Pb}}_{8}$${\mathrm{Cu}}_{2}$${\left({\mathrm{PO}}_{4}\right)}_{6}$$S_{4}$.

**Figure A9:**
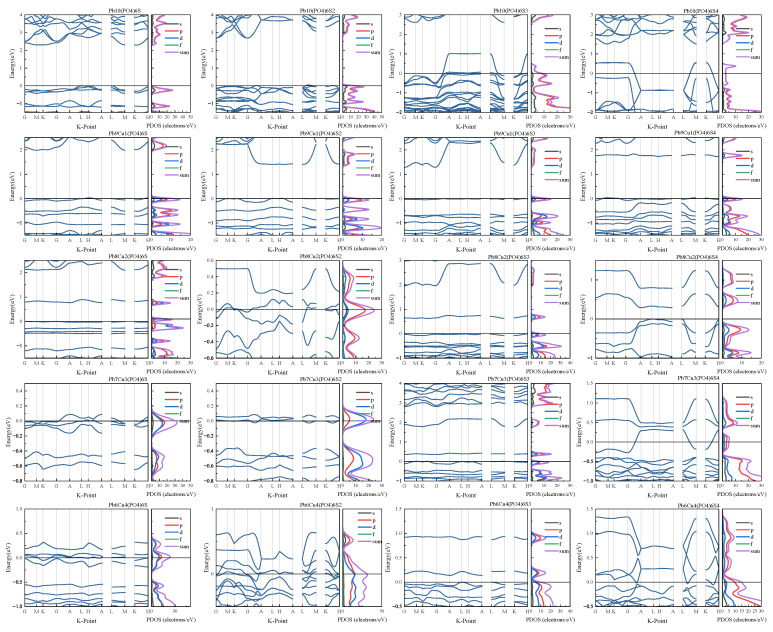
Band structure and PDOS of ${\mathrm{Pb}}_{10-x}$${\mathrm{Cu}}_{x}$${\left({\mathrm{PO}}_{4}\right)}_{6}$$S_{y}$.

**Figure A10:**
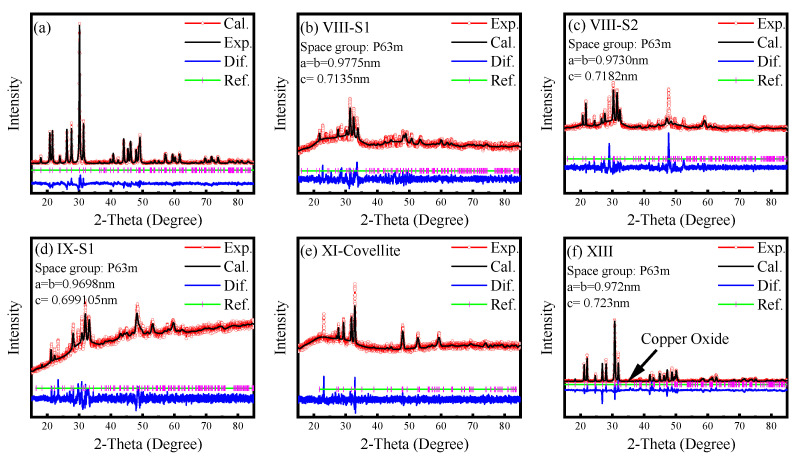
Supplementary XRD patterns of selected samples. (**a**) Representative hydrothermal precursor. (**b**) VIII-S1, (**c**) VIII-S2, (**d**) IX-S1, and (**e**) XI. The red line in (**e**) is the Rietveld refinement fit for Sample XI using a CuS model ($P6_{3}/mmc$). (**f**) Sample XIII, obtained from the oxidation of sample VII.

**Figure A11:**
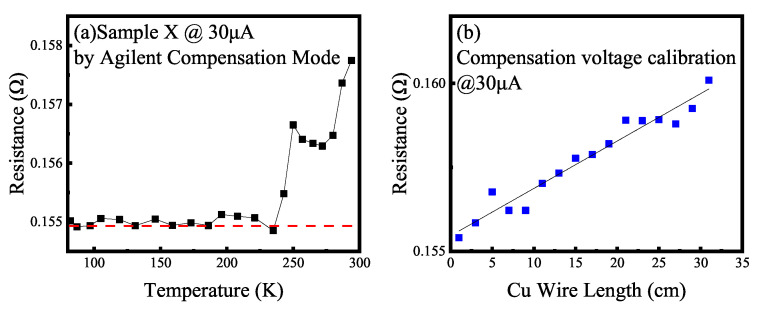
Supplementary resistance measurements for Sample X employing a resistance compensation mode: (**a**) Resistance versus temperature profile. (**b**) Calibration of the system’s offset resistance using copper (Cu) films of varying lengths; this determined offset is subsequently treated as the zero-resistance baseline for the sample measurements.

**Figure A12:**
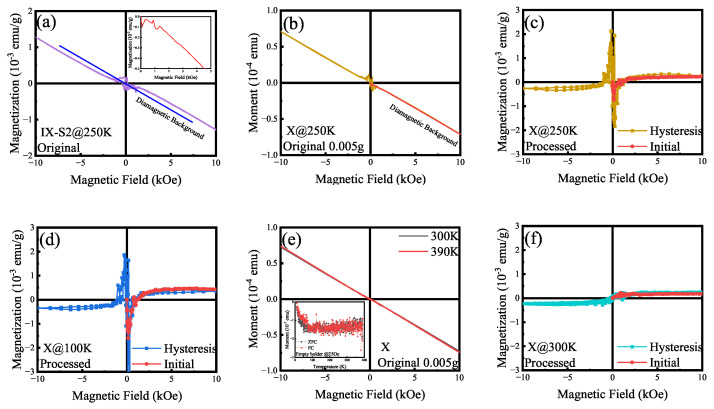
Methodology for processing magnetic data. (**a**,**b**) Raw M-H (magnetization versus magnetic field) data for Samples IX and X, respectively. The diamagnetic background, which is not an intrinsic property of the samples, is indicated. (**c**) Resultant M-H hysteresis loop after the subtraction of the diamagnetic background contribution; this corrected data is also presented in the main text. (**d**) Supplementary M-H loop measured at 100 K. (**e**) M-H loops recorded at 300 K and 390 K. The inset displays the capsule holder employed for these measurements. (**f**) M-H loop at 300 K, exhibiting characteristic soft magnetic behavior.

**Figure A13:**
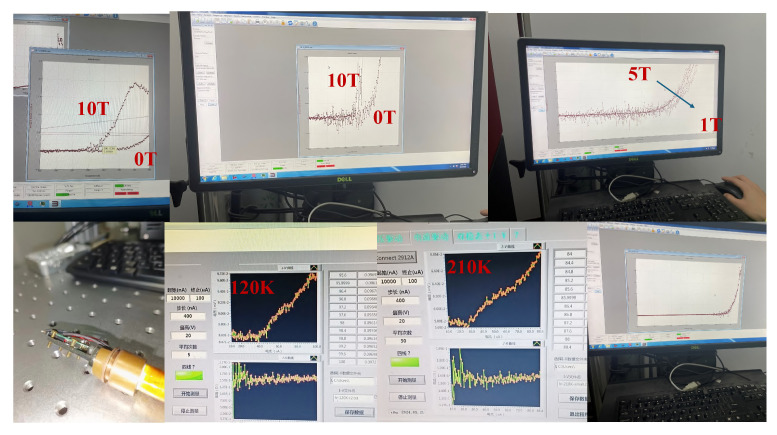
The electrical transport properties were measured across different temperature ranges to prevent sample degradation at elevated temperatures. Specifically, measurements under magnetic fields of 0 T and 10 T were conducted in the temperature range of 2 K to 400 K, whereas measurements for fields between 1 T and 5 T were limited to a range of 2 K to 325 K.

**Figure A14:**
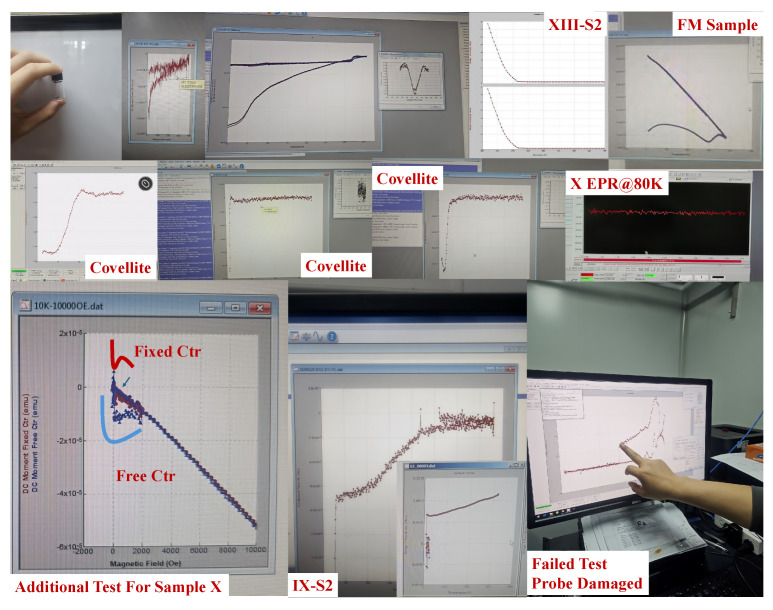
Real-time photographs of the measurements for Sample X, corresponding to data presented in this paper. These images include data from tests where issues were encountered. The brittleness of the pressed powder sample often led to fracturing when attaching the electrical contacts. We conducted multiple repeated measurements on Sample X and confirmed that the general data trends were consistent and reproducible.

**Figure A15:**
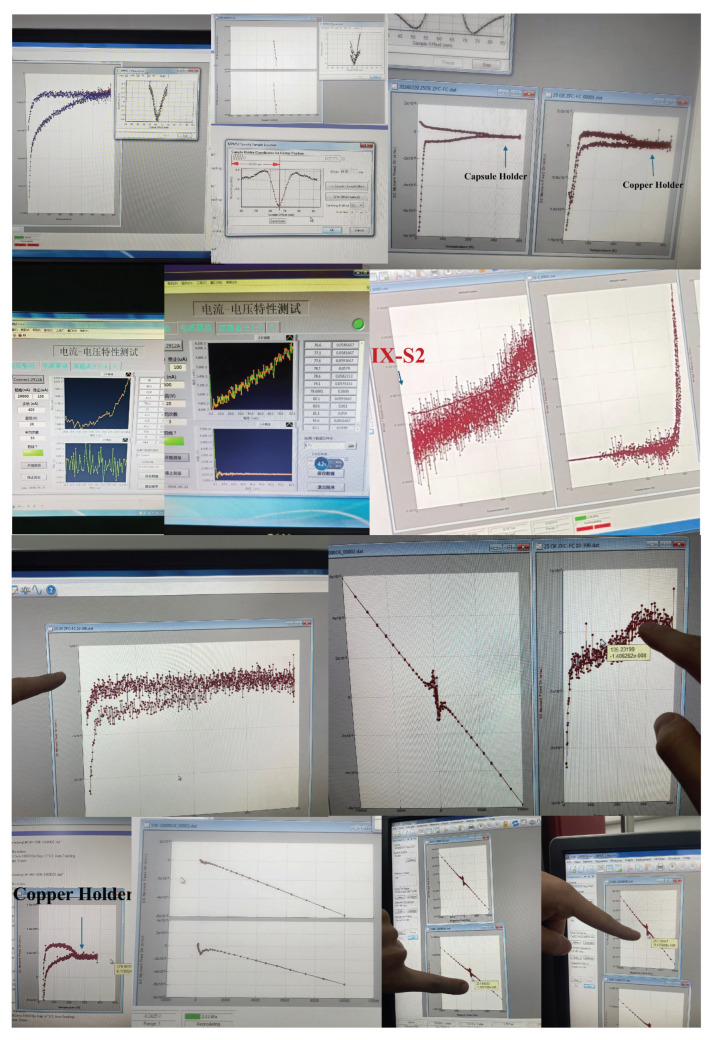
The shown are details from the magnetic and electrical measurements, with images captured directly from the equipment display. To avoid spurious signals, a different sample holder was used for the zero-field-cooled (ZFC) and field-cooled (FC) measurements, which were conducted thoroughly on all samples.

**Figure A16:**
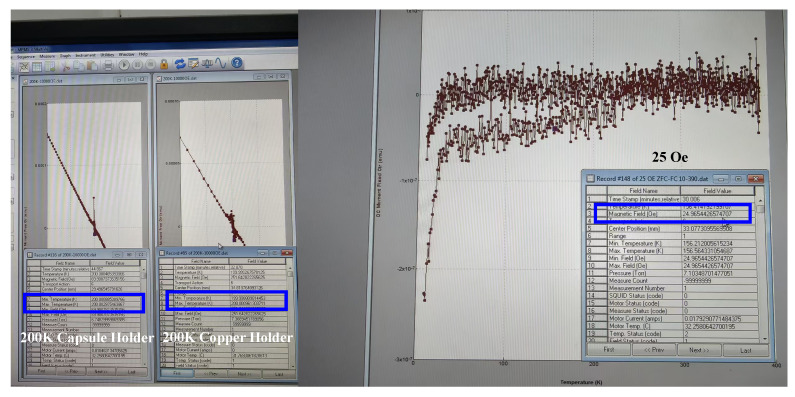
Magnetization details. The blue border emphasizes the temperature and specific magnetic field of the test.

## Data Availability

The original contributions presented in this study are included in the article. Further inquiries can be directed to the corresponding author.

## Appendix A. Details of Syntheses and Challenges

### Appendix A.1. Synthesis of Pb_10−x_Cu_x_(PO_4_)_6_O

Following the procedure for Sample III, we synthesized a series of copper-doped apatite samples using a hydrothermal-calcination method. The lattice constants of these samples were determined through Rietveld refinement. A subset of these samples was subjected to water quenching to investigate the crystallographic differences compared to slowly cooled samples. The relative unit cell volume was calculated with respect to the undoped lead apatite [73]. A clear trend emerges: the unit cell volume decreases nearly linearly with increasing copper concentration [74]. This observation is well-supported by both our experimental data and theoretical calculations. Notably, the quenched samples exhibit a more pronounced lattice contraction.

We have compiled crystallographic data from the existing literature on apatite. By applying the linear relationship established in our study, we have corrected the estimated copper doping levels for these previously reported samples (for which specific doping concentrations were often not disclosed) [75]. Our analysis reveals that, with the exception of the data reported by the Max Planck Institute [49], the majority of synthesized samples appear to be significantly under-doped with copper. Similarly, the reported lattice constants for LK-99 are anomalously large, which could be attributed to either insufficient copper doping or the presence of oxygen vacancies within the one-dimensional channels.

### Appendix A.2. Synthesis of (Pb, Cu)_10_(PO_4_, SO_4_)_6_S_1+x_

A detailed description of our experimental methodology is provided herein. Throughout the experimental process, the atomic ratio of Cu/Pb was controlled within the range of 2/8 to 4/6. Although achieving such high levels of copper incorporation may not always be feasible, efforts were made to maintain an excess of copper precursors. The sequence of precursor addition in the hydrothermal method is of paramount importance. If a mixed solution of ${\mathrm{Cu}}^{2+}$ and ${\mathrm{Pb}}^{2+}$ ions is prepared prior to the addition of ${\mathrm{PO}}_{4}^{3-}$ ions, the synthesis of the apatite phase will invariably fail. Therefore, the experimental procedure prioritizes the initial formation of copper phosphate, accompanied by an adjustment of the solution pH to neutrality. Subsequently, lead phosphate is introduced, followed by the final addition of lead sulfide. It was observed that employing pure, reagent-grade lead sulfide yields superior reaction outcomes compared to using co-precipitated lead sulfide, although the specific underlying reasons for this difference remain unclear at present. Regarding reagent selection, the use of $K_{2}$S (which may include potassium polysulfides) as the soluble sulfide salt provides optimal results and exhibits high reproducibility. Conversely, preparations utilizing sodium sulfide frequently yield insulating materials or semiconductors with exceptionally high electrical resistance. For the synthesis of Sample VII, a critical initial step involves the thorough aging of the precursor solution under vigorous stirring in an aqueous medium for a minimum of 24 h. This aging process is accompanied by a distinct color change of the solution from blue-gray to black-gray, an observation of significant importance. Proceeding directly to the hydrothermal stage without adequate aging will invariably lead to synthetic failure. The correctly synthesized Sample VII should manifest as a gray powder, and its X-ray diffraction (XRD) pattern should be devoid of any impurity peaks.

It is particularly noteworthy that during this initial synthesis stage, the product can exhibit a wide array of colors, including green, light green, blue, white, black, or brown. Consequently, careful color-based selection of the product from this first step is crucial. These color variations are tentatively attributed to subtle differences in solution pH control and the occurrence of unanticipated substitution reactions. During the subsequent, second hydrothermal process, meticulous control over the $S^{2-}$ concentration and hydrothermal parameters (e.g., duration, temperature) is essential. This stage typically requires careful empirical optimization, involving the gradual increase of $S^{2-}$ content and hydrothermal treatment time. The objective is to maintain the sample in a critically balanced, near-transition state, a factor of considerable importance for achieving the desired properties. With the exception of samples intentionally synthesized to investigate the specific roles of CuS, ${\mathrm{Cu}}_{2}$S, or elemental Cu, the majority of samples, including Sample X, underwent a copper removal treatment. This procedure was implemented to mitigate potential misinterpretations or confounding effects arising from theories involving copper sulfide species (e.g., ${\mathrm{Cu}}_{2}$S). For this separation, we employed flotation agents typically used for copper sulfide ores, primarily composed of xanthates and dialkyl dithiophosphates. As a direct consequence of this treatment, despite the incorporation of substantial amounts of copper during the initial synthesis, the XRD patterns of samples like Sample X typically do not exhibit characteristic peaks corresponding to CuS or ${\mathrm{Cu}}_{2}$S phases.

The morphology and elemental composition of representative apatite–sulfide composites were investigated using Scanning Electron Microscopy coupled with energy-dispersive X-ray spectroscopy (SEM-EDS). The analysis revealed that a majority of the synthesized particles exhibited a hexagonal morphology. Notably, a small fraction of particles retained the pristine, rod-shaped structure characteristic of the apatite precursor. However, careful differentiation of the hexagonal structures is warranted, as the presence of copper sulfide (CuS), which also crystallizes in a hexagonal system, can lead to morphological ambiguity and potential misinterpretation.

A summary of the critical parameters in the synthesis process is as follows:

1. It is imperative that the synthesis commences with copper(II) phosphate, $Cu_{3}{\left(PO_{4}\right)}_{2}$, as the primary precursor. The introduction of copper in the form of copper sulfide (CuS) is to be avoided. This initial step is of fundamental importance. Lee likewise posits that copper(I) phosphide ($Cu_{3}P$) is a critical starting material.
2. A stoichiometric excess of copper should be ensured during the synthesis. This promotes the formation of the desired composite of $Cu_{3}{\left(PO_{4}\right)}_{2}$, lead(II) phosphate ($Pb_{3}{\left(PO_{4}\right)}_{2}$), and lead(II) sulfide (PbS). Any resultant excess copper sulfide can be subsequently removed through mineral processing techniques.
3. A two-step synthesis protocol is mandatory. The initial step involves the formation of the apatite framework, which is then subjected to sulfur doping in the second step. A single-step approach is ineffective as it precludes the successful incorporation of dopants into the apatite structure, leading instead to a product mixture dominated by copper sulfides and lead phosphates.
4. The aging process holds significantly greater importance than the hydrothermal treatment. The temperature of the hydrothermal stage should not be excessively high. Close observation of the sample’s color evolution during the aging process is a critical indicator.
5. Achieving precise control over the copper doping concentration is challenging. Consequently, a high-throughput experimental approach is recommended to facilitate the screening of numerous samples and identify those with the desired properties.

### Appendix A.3. Rapid Screening

Initial visual assessment of sample color is a crucial first step for the efficient progression of experiments. Sample X, for instance, is characterized by its black color and lack of metallic luster. The presence of covellite impurities often imparts a partial brown or blue coloration to the sample, allowing for a preliminary rapid screening based on this observation.

For a more comprehensive evaluation, most samples can be rapidly screened using X-ray diffraction (XRD) and electron paramagnetic resonance (EPR) spectroscopy. Structural refinement of the XRD data, typically Rietveld refinement, is performed; a key indicator of a promising sample is a significant contraction in its lattice parameters. Samples exhibiting substantial quantities of covellite and galena phases, as identified by XRD, are generally deemed unsuitable for further investigation.

EPR spectroscopy serves as an effective tool for the rapid identification of potentially useful samples. At room temperature, target materials are expected to exhibit weak paramagnetism. Any diamagnetic signal observed under these conditions is primarily attributed to the presence of PbS or undoped apatite or phosphate. Consequently, if a sample demonstrates purely diamagnetic behavior in EPR measurements at room temperature, it is typically excluded from further consideration. The application of EPR thus facilitates the swift identification of samples possessing the desired ferromagnetic characteristics, ensuring an efficient screening process and enabling a more effective optimization of the synthesis protocol. It is patently inadequate to draw definitive conclusions in this research based solely on an overly simplified synthesis.

Magnetic measurement takes priority over electrical measurement. In the MPMS-3 device, it takes 30 min to complete the screening of a sample. Specifically, zero field cooling to 4 K and applying a magnetic field of 10 to 20 Oe can directly determine whether the sample has effective diamagnetism.

### Appendix A.4. Challenges in Repetitive Experiments

Hundreds of samples were synthesized, of which a selection demonstrating typical characteristics and superior performance is presented herein. Furthermore, results from selected samples displaying non-ideal resistive behavior are also presented. Although diamagnetic transitions were observed in conjunction with resistive changes, the presumably low-volume fraction of the superconducting phase in these heterogeneous samples may lead to complex and unpredictable current distribution. Notably, during the attempted replication of Sample XIII, an anomalous sharp upturn in resistance was observed below 4 K, a phenomenon not previously documented in replication efforts.

It must be emphasized that the synthesis protocol described herein currently suffers from limitations in reproducibility. This is principally attributed to the difficulties encountered in managing the deep doping of sulfur (S). Attaining precise control over the S doping concentration and the effective suppression of impurities requires considerable further investigation to achieve a breakthrough. Concurrently, the incorporation of copper (Cu) also revealed significant uncontrolled variables during the experimental process. Even with the intentional addition of significant Cu quantities, the formation of unintended phases, indicative of unsuccessful doping, persisted in some instances.

Interestingly, the formation of sulfides is not the exclusive outcome of reactions involving apatite or its precursors. In this study, building upon formulation VII (nominal Pb:Cu atomic ratio of 8:2), a composite material consisting of coexisting apatite and Palmierite ($K_{2}$Pb(SO${\left({\mathrm{SO}}_{4}\right)}_{2}$) phases was synthesized. The potassium (K) content in Palmierite originates from $K_{2}$S and $K_{3}$${\mathrm{PO}}_{4}$, while its sulfate (${\mathrm{SO}}_{4}^{2-}$) groups are believed to be entirely formed through a substitution pathway where sulfur (from $K_{2}$S) effectively replaces phosphorus (present in $K_{3}$${\mathrm{PO}}_{4}$), ultimately forming the sulfate anions within the Palmierite structure. The resistance–temperature (R-T) characteristics and magnetic properties of this composite were investigated. To discern the intrinsic contributions from the synthesized phases, potential ${\mathrm{Cu}}_{2}$S impurities were selectively removed via a solution-based leaching method. The key electrical and magnetic signatures persisted post-treatment, largely ruling out significant interference from ${\mathrm{Cu}}_{2}$S. However, a distinct semiconducting material is present within the mixture, and its removal during the fabrication process was not feasible. In initial investigations, the magnetic transition temperature was identified at 323 K, whereas the resistive transition occurred above 270 K. While the abrupt change in resistance can be attributed to the phase transition of ${\mathrm{Cu}}_{2}$S, the transition observed near 323 K currently lacks a definitive explanation. Furthermore, subsequent studies by Lee and his team have consistently reported a transition around 323 K [5,76]; however, the non-smooth physical phenomena associated with this transition have not yet been thoroughly investigated.

Considering that achieving superconductivity in variant apatite may necessitate significant crystal lattice contraction, and given the suboptimal interfacial lattice matching between galena or covellite and variant apatite, future investigations could employ Mo$S_{2}$ as a buffer layer.

### Appendix A.5. Challenges on the Uncertainty of Structures

The superconducting transition in our material is consistently observed via magnetic susceptibility measurements. However, the manifestation of zero resistance in electrical transport measurements proves to be elusive, achieved in less than 5% of our synthesized samples. This low success rate is attributed to significant challenges in controlling the phase purity and microscopic homogeneity of the material, necessitating high-throughput synthesis to isolate optimal samples.

The primary source of this irreproducibility arises from two key areas. First, the initial hydrothermal synthesis step yields inconsistent products—as evidenced by significant color variations among batches—despite stringent control of reaction conditions. This variability dictates that only a select portion of samples is viable for the subsequent sulfurization process.

Second, achieving controlled copper doping within the apatite lattice is fundamentally challenging. The high thermodynamic stability of copper sulfide (CuS) creates a competing reaction that inhibits the desired incorporation of copper, an issue that persists despite adjustments to reactant sequencing and pH. Moreover, a significant discrepancy exists between macroscopic stoichiometry and microscopic reality. While Density Functional Theory (DFT) calculations confirm the thermodynamic feasibility of doping, they also predict that at the atomic level, phase separation is energetically favored over uniform dopant distribution. For example, our calculations indicate that a mixture of copper-rich and copper-poor domains (e.g., ${\mathrm{Pb}}_{8}$${\mathrm{Cu}}_{2}$${\left({\mathrm{PO}}_{4}\right)}_{6}$S+${\mathrm{Pb}}_{10}$${\left({\mathrm{PO}}_{4}\right)}_{6}$S) is more stable than a homogeneous ${\mathrm{Pb}}_{9}$${\mathrm{Cu}}_{1}$${\left({\mathrm{PO}}_{4}\right)}_{6}$S structure [38].

In the initial phase of our research, we identified the optimal substitution sites for copper in ${\mathrm{Pb}}_{10-x}$${\mathrm{Cu}}_{x}$${\left({\mathrm{PO}}_{4}\right)}_{6}$$S_{y}$ for doping concentrations where x=1−4 in Appendix B. We also qualitatively described the contraction of the unit cell volume as the concentration of persulfide (increasing *y*) in the one-dimensional channel increased. Currently, the precise chemical formula responsible for the observed superconductivity in the samples remains undetermined. Therefore, we conducted Electron Probe Microanalysis (EPMA) point quantification, as detailed in Table A1, to identify several potential stoichiometric ratios.

**Table A1:** Stoichiometric ratios of variant apatite and covellite.

Apatite					Covellite		
Pb	Cu	${\mathrm{PO}}_{4}$	${\mathrm{SO}}_{4}$	S	Cu	Pb	S
9	1	6	0	2	0.95	0.05	1.16
8	2	5.5	0.5	2–3			
7	3	5	1	2–4			
6	4	4	2	3–4			

This predicted microscopic inhomogeneity presents a major analytical challenge. Although techniques such as Electron Probe Microanalysis (EPMA) can confirm the elemental composition of individual grains, they lack the resolution to identify the precise local structure and stoichiometry of the phase responsible for superconductivity, and it is usually very difficult in early research [77]. Resolving this atomic-scale heterogeneity is beyond the scope of the current study but is identified as the principal cause of the weak and irreproducible superconducting signal.

## Appendix B. Density Functional Theory Calculations

To complement the experimental findings, we conducted DFT calculations to gain qualitative insights into the effects of Cu substitution and S doping. Detailed results and discussions are presented in the appendix.

### Appendix B.1. Electronic Structure of Variant Apatite

We conducted an in-depth investigation using the Vienna Ab initio Simulation Package for first-principles calculations [78]. In this process, we adhered to the framework of the Generalized Gradient Approximation and employed the Perdew Burke Ernzerhof functional [79] to determine the exchange correlation potential. To ensure the accuracy of our calculations, we comprehensively relaxed the atomic positions and lattice constants of each system until the maximum force acting on each atom converged below 0.01 eV/Å, while the convergence criterion for the total energy was rigorously set to $10^{-7}$ eV. When setting up the wave functions, we selected an energy cutoff value of 630 eV and utilized a 3 × 3 × 3 Monkhorst-Pack K-point grid centered at the Γ-point for momentum space integration. During the calculation of the electronic structure, we employed the PBE + U method to correct for the Coulomb repulsion in transition metal atoms Cu with an effect U = 4.0 eV [80].

These stoichiometries were then subjected to more refined calculations. The calculations did not account for the potential effects of ${\mathrm{SO}}_{4}$ substitution. The specific crystal chemical formulas used in the computations were ${\mathrm{Pb}}_{9}$Cu${\left({\mathrm{PO}}_{4}\right)}_{6}$$S_{2}$, ${\mathrm{Pb}}_{8}$${\mathrm{Cu}}_{2}$${\left({\mathrm{PO}}_{4}\right)}_{6}$$S_{2}$, ${\mathrm{Pb}}_{8}$${\mathrm{Cu}}_{2}$${\left({\mathrm{PO}}_{4}\right)}_{6}$$S_{3}$, ${\mathrm{Pb}}_{7}$${\mathrm{Cu}}_{3}$${\left({\mathrm{PO}}_{4}\right)}_{6}$S$S_{3}$, ${\mathrm{Pb}}_{7}$${\mathrm{Cu}}_{3}$${\left({\mathrm{PO}}_{4}\right)}_{6}$$S_{4}$, ${\mathrm{Pb}}_{6}$${\mathrm{Cu}}_{4}$${\left({\mathrm{PO}}_{4}\right)}_{6}$$S_{3}$, and ${\mathrm{Pb}}_{6}$${\mathrm{Cu}}_{4}$${\left({\mathrm{PO}}_{4}\right)}_{6}$$S_{4}$.

The structure of ${\mathrm{Pb}}_{9}$Cu${\left({\mathrm{PO}}_{4}\right)}_{6}$$S_{2}$ has the highest formation energy, as shown in Figure A5a. The electronic structure of ${\mathrm{Pb}}_{9}$Cu${\left({\mathrm{PO}}_{4}\right)}_{6}$$S_{2}$ exhibits a semiconductor characteristic, with a band gap of about 0.313 eV; however, the spin-up band gap is about 1.932 eV. Especially for the spin-up part at the Γ point, the spin gap is only about 0.017 eV. However, after considering the spin-orbit coupling (SOC), the spin-up gap at the Γ point enlarges to 0.047 eV. But the most important effect of the SOC is that the LUMO of the spin-down part moves entirely up to about 0.22 eV. The magnetic moment for the Cu atom is 0.312 $\mu_{B}$, while the two S atoms are about 0.133 and 0.175 $\mu_{B}$. The most important results for the ${\mathrm{Pb}}_{9}$Cu${\left({\mathrm{PO}}_{4}\right)}_{6}$$S_{2}$ structure are that the doped Cu atom and its two nearest neighbor S atoms form a quasi one-dimensional channel, as shown in the two-dimensional magnetic map. It can be observed from the figure that in a quasi one-dimensional channel, the Cu atom has four lobes, while the S atoms’ pz orbitals interact with each other.

As for the second-highest formation energy in the ${\mathrm{Pb}}_{8}$${\mathrm{Cu}}_{2}$${\left({\mathrm{PO}}_{4}\right)}_{6}$$S_{2}$ system (as shown in Figure A5c), which shows a half-metallic feature [81], the spin-up band shows a band gap of approximately 0.264 eV, while the spin-down band crosses the Fermi level. The most important feature for the spin-up band is that there are two quite flat bands, which are about 0.22 eV above the Fermi level from the Γ-A to the A-M region, and about 0.52 eV above the Fermi level from the Γ-Γ point region. Moreover, the peak at the H point above the Fermi level becomes lower after considering the SOC, and all the flat bands become lower in energy. These electronic structures may be related to the one-dimensional structure as shown in Figure A5 We should point out that the two doped Cu atoms have different magnetic moments; one is about 0.387 $\mu_{B}$, while the other one is only about 0.053 $\mu_{B}$. And the two doped S atoms have similar magnetic moments, 0.231 and 0.226 $\mu_{B}$.

As for the third-highest formation energy, the ${\mathrm{Pb}}_{8}$${\mathrm{Cu}}_{2}$${\left({\mathrm{PO}}_{4}\right)}_{5}$$S_{3}$ shows a very tiny band gap of about 0.0168 eV, but with a spin-up band gap of about 0.473 eV and a spin-down band gap of about 0.551 eV. Its electronic structure is very similar to that of ${\mathrm{Pb}}_{7}$${\mathrm{Cu}}_{3}$${\left({\mathrm{PO}}_{4}\right)}_{6}$$S_{3}$, but the latter has a larger band gap of 0.075 eV, with the spin-down gap of 0.543 eV and the spin-up gap of about 0.390 eV. As for other configurations, such as ${\mathrm{Pb}}_{6}$${\mathrm{Cu}}_{4}$${\left({\mathrm{PO}}_{4}\right)}_{6}$$S_{3}$, and ${\mathrm{Pb}}_{6}$${\mathrm{Cu}}_{4}$${\left({\mathrm{PO}}_{4}\right)}_{6}$$S_{4}$, all show normal metallic behavior.

DFT studies have demonstrated a wide range of potential properties in variant apatite and corroborated the experimental findings of its soft magnetic behavior. The calculations were predominantly utilized for high-throughput screening and for elucidating the complex properties induced by doping with non-metallic elements. Although determining superconductivity directly from the electronic structure is challenging, the observed magnetic properties in apatite are not inconsistent with superconductivity and may, in fact, be synergistic. In such a scenario, magnetic fluctuations can mediate attractive interactions between electrons [82,83], a mechanism that typically leads to unconventional superconductivity with pairing symmetries beyond the s-wave.

### Appendix B.2. High-Throughput Screening of Apatite

We employed CASTEP [84] to investigate the crystal structure resulting from excessive sulfur (oxygen) doping and to identify potentially favorable outcomes. The calculations were performed using the LDA functional [85]. The resulting lattice constants and formation energies were used solely for a qualitative analysis of the optimal configurations and the corresponding changes in unit cell volume. The Hubbard *U* correction was not considered in these calculations. The atomic positions and lattice constants of each system were fully relaxed until the maximum force on any atom was less than 0.01 eV/Å, with a strict total energy convergence of $10^{-7}$ eV. An energy cutoff of 630 eV was applied.

A challenge arises from the relatively disordered nature of doping sites within the apatite structure, which contrasts with the layered arrangement characteristic of perovskite systems and instead exhibits a more chain-like configuration. The formation of a reaction pathway that can be calculated is consistent with the experiment, which is PbS + ${\mathrm{Pb}}_{3}$${\left({\mathrm{PO}}_{4}\right)}_{2}$ + ${\mathrm{Cu}}_{3}$${\left({\mathrm{PO}}_{4}\right)}_{2}$. For a sulfur doping level of **y<2**, the crystal lattice exhibits a moderate shrinkage, a finding that is consistent with experimental results. However, when **y>2**, adhering to the proposed ${\mathrm{Pb}}_{10-x}$${\mathrm{Cu}}_{x}$${\left({\mathrm{PO}}_{4}\right)}_{6}S_{y}$ stoichiometry would theoretically lead to a rapid expansion of the unit cell volume. Guided by quantitative results from EPMA, we therefore modeled an alternative mechanism involving the substitution of phosphate (${\mathrm{PO}}_{4}^{3-}$) by sulfate (${\mathrm{SO}}_{4}^{2-}$) groups, without optimizing for a specific crystallographic site. In contrast to the y=1 case, this model shows that the ${\mathrm{SO}}_{4}^{2-}$ for ${\mathrm{PO}}_{4}^{3-}$ substitution induces a significant lattice contraction, which aligns with macroscopic experimental observations.

We further illustrate the Cu occupation sites with the lowest total energy for various Cu and S doping concentrations. It is noteworthy that for Cu doping levels of x=3 and x=4, as the S doping concentration increases, the calculated Cu occupation sites change from a 2×(Pb2)−1×(Pb1) configuration and an adjacent 4×(Pb2) configuration to a 3×(Pb2) configuration and a symmetric 4×(Pb2) configuration, respectively.

Additionally, we present the calculated band structure and density of states (DOS) for the non-spin-polarized case in Figure A9. The diverse physical properties of these apatite variants are strongly correlated with the co-doping concentrations of Cu and S. It should be emphasized that, due to the clustering and instability of copper during the doping process, we still cannot clarify the specific and detailed doping ratio. All calculations tend to focus on high-throughput screening rather than providing a clear phase and result [86,87].

## Appendix C. Detail in Lattice Parameters

The results of the Rietveld refinement performed on the XRD data are displayed in Figure A10.

The lattice constants for all samples are presented in Table A2, along with their corresponding crystal structures. It is important to note that the results from X-ray diffraction (XRD) refinement represent a macroscopic average of the bulk material. This technique does not resolve local structural variations that may arise from specific doping configurations.

For context, the oxygen apatite host material exhibits some level of electrical conductivity. As an illustrative example, we performed four-probe and Hall effect measurements on Sample III (paramagnetic) at room temperature (300 K). The sample showed a resistivity (ρ) of 3.17 × $10^{4}$ Ω·cm, a carrier mobility (μ) of 9.35 ${\mathrm{cm}}^{2}/(V\cdot$s), a carrier concentration (*n*) of $2.11\times 10^{13}$ ${\mathrm{cm}}^{-3}$, and a Hall coefficient ($R_{H}$) of $-2.96\times 10^{5}$ ${\mathrm{cm}}^{3}$/C. However, given its very high room-temperature resistivity, the material is classified as an insulator.

Since the sample did not consistently crystallize in the $P6_{3}/m$ space group, its structure was refined using the P1 space group, which yielded a comparable crystallite size.

**Table A2:** Lattice parameters and properties.

Order	a = b (Å)	c (Å)	Magnetic Pro.	Electrical Pro.	Impurities
I	9.845	7.426	DM	Insulator	
II	9.846	7.425	DM + SM	Insulator	
III	9.84	7.421	PM	Insulator	
IIII	9.837	7.419	PM + SM	Insulator	
V	9.835	7.405	PM	Insulator	
VI	9.833	7.395	PM + ME	Insulator	
VII	9.851	7.401	PM	Metallic	Covellite
VIII-S1	9.775	7.135	-	Metallic	Covellite
VIII-S2	9.73	7.182	-	Metallic	Covellite
IX-S1	9.698	6.991	ME	Metallic	Covellite + Galena
IX-S2	9.696	7.012	ME	Metallic	Covellite
X	9.699	7.001	ME	SC1	Galena + Covellite
XI	Covellite		ME	SC2	Galena
XII	Phosphate		FM	Semiconductor	Covellite + galena
XIII	9.72	7.23		MIT	Oxide
XIII	9.783	7.315	PM	semiconcudtor	Oxide and sulfate
	a (Å)	b (Å)	c (Å)	Space Group	
X	9.70	9.675	0.699	P1	
	α	β	γ	Lattice Type	
	86.32°	89.17°	111.25°	Triclinic	

PM: Paramagnetic; DM: Diamagnetic; SM: Soft Magnetic; ME: Meissner effect; FM: Ferromagnetic; SC1: Superconductivity in Apatite; SC2: Superconductivity in Covellite; MIT: Metal Insulator Transition.

## Appendix D. Detail in Test by Agilent

Prior to electrical characterization, the samples were pressed into blocks. For the four-probe resistance measurements, each block was securely mounted in a standard sample holder designed to minimize vibrational noise and maintain stable electrical contact with the electrodes across the varying temperature range, thereby accommodating thermal expansion and contraction effects.

Concurrently, the temperature-dependent resistance of the samples was further investigated (or independently measured) using Agilent test instrumentation. A voltage compensation technique was employed during these measurements as shown in Figure A11. This approach establishes a consistent baseline or reference point (often referred to as a “fixed initial resistance” in raw data conversion) for the accurate calculation of the sample’s resistance by systematically accounting for or nullifying system offsets.

## Appendix E. Detail in Secondary Processing

Given the low magnitude of the magnetic signals in this study, the raw magnetic data underwent post-acquisition processing as shown in Figure A12a,b. Consequently, it is pertinent to detail our data processing methodology and the resultant outcomes. During the magnetic measurements (e.g., M-H curve acquisition), gelatin capsules were employed as sample holders. These capsules and lead compound in impurities such as galena exhibit a faint diamagnetic background. All authors affirm the authenticity of the raw data presented herein. Only the magnetic data have been subjected to post-acquisition processing.

## Appendix F. Authenticity of the Data

First authors H. Wang and H. Wu attest to the authenticity of dates presented herein. Furthermore, to address any potential scrutiny regarding data veracity, we have supplemented this work with a depiction of the on-site experimental conditions during testing for full transparency in Figure A13, Figure A14, Figure A15, Figure A16.
